# Cajaninstilbene Acid Ameliorates Cognitive Impairment Induced by Intrahippocampal Injection of Amyloid-β_1–42_ Oligomers

**DOI:** 10.3389/fphar.2019.01084

**Published:** 2019-09-24

**Authors:** Li-Sha Wang, Xue Tao, Xin-Min Liu, Yun-Feng Zhou, Meng-Di Zhang, Yong-Hong Liao, Rui-Le Pan, Qi Chang

**Affiliations:** ^1^Institute of Medicinal Plant Development, Chinese Academy of Medical Sciences and Peking Union Medical College, Beijing, China; ^2^National Key Laboratory of Human Factors Engineering and the State Key Laboratory of Space Medicine Fundamentals and Application, China Astronaut Research and Training Center, Beijing, China

**Keywords:** Alzheimer’s disease, amyloid-β oligomer, cajaninstilbene acid, cognition, microglia, astrocyte, glutamate, N-methyl-D-aspartate receptor

## Abstract

Amyloid-β_1–42_ (Aβ_1–42_) oligomers play an important role at the early stage of Alzheimer’s disease (AD) and have been a vital target in the development of therapeutic drugs for AD. Cajaninstilbene acid (CSA), a major bioactive stilbene isolated from pigeon pea (*Cajanus cajan*) leaves, exerted the neuroprotective property in our previous studies. The present study utilized a validated mouse model of early-stage AD induced by bilateral injection of Aβ_1–42_ oligomers into hippocampal CA1 regions (100 pmol/mouse) to investigate the cognitive enhancing effects of CSA and the underlying mechanism, by a combination of animal behavioral tests, immunohistochemistry, liquid chromatography-tandem mass spectrometry analysis, and Western blot methods. Intragastric administration of CSA (7.5, 15, and 30 mg/kg) attenuated the impairment of learning and memory induced by Aβ_1–42_ oligomers. CSA stimulated Aβ clearance and prevented microglial activation and astrocyte reactivity in the hippocampus of model mice. It also decreased the high levels of Glu but increased the low levels of GABA. In addition, CSA inhibited excessive expression of GluN2B-containing NMDARs and upregulated the downstream PKA/CREB/BDNF/TrkB signaling pathway. These results suggest that CSA could be a potential therapeutic agent at the early stage of AD.

## Introduction

Alzheimer’s disease (AD) is a main form of dementia in the elderly and characterized by the accumulation of senile plaques which are composed of fibrillar amyloid-β peptide (Aβ) (Cohen and Calkins, 1959). Recent experiments suggest that Aβ oligomers are more toxic than fibrils (Dahlgren et al., 2002; Balducci et al., 2010; DaRocha-Souto et al., 2011) and have already been detected in AD-affected human brains, 7PA2 cells and Tg2576 mice(Walsh et al., 2002; Gong et al., 2003; Lesne et al., 2006), inducing AD-type pathology (Walsh et al., 2002; De Felice et al., 2008; Jin et al., 2011). Based on the important role of Aβ oligomers in Alzheimer’s dementia, the Aβ oligomers hypothesis was proposed (Klein, 2002). Aβ oligomers form readily at low concentrations, whereas significant higher levels of Aβ monomer are required to foster protofibrils formation, suggesting that Aβ oligomers would be first to appear and involve in early-stage AD(Klein, 2002). Aβ_1–42_ aggregates more rapidly than Aβ_1–40_ (Snyder et al., 1994) and is more toxic than Aβ_1–40_ (Sultana et al., 2009). Mutations in amyloid precursor protein, presenilin-1 or presenilin-2 have been shown to increase the production of Aβ_1–42_ and lead to early onset of AD (Selkoe, 2001). Therefore, Aβ_1–42_ oligomers play an important role at the early stage of AD.

Currently available treatments for AD are symptomatic and do not decelerate or prevent AD progression (Herrmann et al., 2011). In light of past failures of anti-Aβ antibodies (solanezumab, bapineuzumab, and gantenerumab) in phase III trials, the AD community has increasingly embraced the idea that disease modification depends on earlier initiation of treatment (Mullard, 2016). Animal models with excess Aβ burden in the brain are vital tools for modeling AD states. Pathogen-induced AD models produced by direct injection of Aβ into the brain can not only show AD-like behavioral abnormalities but also exhibit Aβ pathology to some extent (Kim et al., 2016). Compared with transgenic models, the Aβ-injected animal models are economical, time-saving, and allow researchers to control different conditions to focus on the downstream pathology of Aβ abnormalities. Accumulating studies have adopted the Aβ_1–42_ oligomers-injected animal models to screen therapeutic agents for early-stage AD.

After intracerebral injection, Aβ_1–42_ oligomers have been shown to impair animal recognition memory in the object recognition task (Figueiredo et al., 2013), spatial learning, and memory in Morris water maze (MWM) (Zhang et al., 2014), working memory in Y maze (Moon et al., 2011), and fear-aggravated memory in passive avoidance test (Choi et al., 2011). The underlying mechanism involves the abnormal status of neurons, astrocytes and microglia (Choi et al., 2011; Ledo et al., 2016; Kasza et al., 2017; Chun et al., 2018). Activated microglia release proinflammatory mediators which influence the efficiency of astrocytic glutamate (Glu) uptake (Zhao et al., 2004). Aβ also downregulates Glu uptake capacity of astrocytes (Matos et al., 2008) and induces the release of astrocytic and microglial Glu (Noda et al., 1999; Talantova et al., 2013). The inhibitory amino acid neurotransmitter γ-aminobutyric acid (GABA) synthesized from Glu contributes to the progression of AD (Bai et al., 2015). Dyshomeostasis of Glu and GABA cause neuronal damage through their downstream receptors.

Among Glu receptors, N-methyl-D-aspartate receptors (NMDARs) are the most important channels mediating Glu excitotoxicity due to their widespread distributions in the central nervous system. The majority of NMDARs are tetrameric complexes containing two obligatory GluN1 subunits and two regulatory GluN2A-D subunits (Cull-Candy et al., 2001). Recent evidence shows that alternation of NMDARs activity plays a vital role in Aβ_1–42_ oligomers-induced neurotoxicity. Low molecular weight fractions of Aβ_1–42_ oligomers induce internalization of GluN1 in cultured hippocampal neurons (Figueiredo et al., 2013). Aβ_1–42_ oligomers significantly reduce synaptic GluN1, GluN2A and GluN2B expression in primary hippocampal neurons (Geng et al., 2013), but activate extrasynaptic GluN2B (Li et al., 2011; Talantova et al., 2013). The activation of extrasynaptic NMDARs contributes to dephosphorylation of cAMP response element binding protein (CREB) (Hardingham et al., 2002), a transcription factor required for long-term memory and neuronal survival and activated by cAMP-dependent protein kinase (PKA). CREB shut-off suppresses the expression of the gene encoding brain-derived neurotrophic factor (BDNF) and leads to less BDNF binding to tropomyosin receptor kinase B (TrkB).

Cajaninstilbene acid (CSA), a main bioactive stilbene from pigeon pea [*Cajanus cajan* (L.) Millsp.] leaves, possesses many bioactivities, including anti-bacterial (Kong et al., 2010), anti-tumor (Fu et al., 2015), anti-osteoporosis (Sun et al., 2019), anti-oxidative (Wu et al., 2011), anti-inflammatory (Patel and Bhutani, 2014; Huang et al., 2016; Schuster et al., 2016), and neuroprotective (Jiang et al., 2014; Liu et al., 2014a) effects. It is reported that stilbenes containing extract-fraction from *Cajanus cajan* (L.) leaves (sECC) ameliorated Aβ_25–35_-induced cognitive impairment (Ruan et al., 2009). As a component in sECC, CSA protected corticosterone-induced impairment in PC12 cells through the inhibition of oxidative stress, endoplasmic reticulum stress-mediated and mitochondrial apoptotic pathways in our previous studies (Jiang et al., 2014; Liu et al., 2014a). In addition, some stilbenes, including resveratrol (Wang et al., 2017), trans-2,4-dimethoxystibene (Ruan et al., 2010a; Ruan et al., 2010b), rhapontigenin (Misiti et al., 2006) and piceatannol (Kim et al., 2007), with similar chemical structures of CSA were reported to have protective effects against Aβ neurotoxicity. Therefore, it is hypothesized that CSA could ameliorate cognitive impairment and be a potential therapeutic agent at the early stage of AD.

To our knowledge, there has been no *in vivo* publication reported the cognitive effects of CSA so far. The present study was conducted to evaluate the cognitive enhancing effects of CSA using a validated mouse model of early-stage AD induced by intrahippocampal injection of Aβ_1–42_ oligomers. The underlying mechanism was explored by monitoring the status of neurons, astrocytes and microglia, detecting the intrahippocampal amount of Aβ, measuring the homeostasis of Glu and GABA, and determining the expression of GluN1 and GluN2B subunits and downstream PKA/CREB/BDNF/TrkB signaling pathway.

## Materials and Methods

### Preparation of Aβ_1–42_ Oligomers

Aβ_1–42_ oligomers were generated as the previously described method (Stine et al., 2003; Fa et al., 2010). Briefly, Aβ_1–42_ (A9810, Sigma, USA) was initially dissolved in 1,1,1,3,3,3-hexafluoro-2-propanol (HFIP, 105228, Sigma, USA) to a final concentration of 1 mM and incubated at room temperature for 2 h. The peptide solution was aliquoted and dried in the fume hood. Traces of HFIP was removed under vacuum in a speedvac (Labconco, USA), and the thin clear peptide film was stored over desiccant at −80ºC. For aggregation, the aliquoted peptide film was dissolved in dimethyl sulfoxide (DMSO) to 5 mM. The peptide in DMSO was diluted directly into phosphate buffered saline (PBS, 1×) at 100 μM and incubated at 4 ºC for 16 h to make the oligomeric form of Aβ_1–42_. Following incubation, the solution was centrifuged at 14,000 × g for 10 min at 4 ºC. Then the supernatant was transferred to a new tube and diluted with the same volume of PBS to a final concentration of 50 μM before stereotactic injections. The Aβ_1–42_ oligomers preparations were routinely characterized by atomic force microscopy (AFM)(Stine et al., 1996) and Western blot using anti-Aβ antibody 6E10 (Lambert et al., 1998) (**Table 1**).

**Table 1:** Antibodies used in this study.

Primary antibodies	Clonality	Source	Dilution	Molecular Wt./kDa	Origin
β-Amyloid, 1-16 (6E10)	M	Mouse	1:2,000/WB; 1:50/IF	4	Biolegend (#803014) (Previously Covance catalog# SIG-39300)
GFAP	P	Rabbit	1:1,000/IF	–	Abcam(ab7260)
Iba1	P	Goat	1:250/IF	–	Abcam(ab5076)
GluN1	M	Rabbit	1:1,000/WB	120	CST(#5704)
GluN2B	M	Rabbit	1:1,000/WB	190	CST(#4212)
PKA c-α	P	Rabbit	1:1,000/WB	42	CST(#4782)
pCREB (Ser133)	M	Rabbit	1:1,000/WB	43	CST(#9198)
CREB-1	M	Rabbit	1:1,000/WB	43	CST(#9197)
BDNF	M	Rabbit	1:3,000/WB	14	Abcam(ab108319)
TrkB	M	Rabbit	1:1,000/WB	90, 140	CST(#4603)
β-actin	M	Rabbit	1:5,000/WB	43	ABclonal(AC026)
Secondary antibodies			Dilution	Conjugate	Origin
Goat anti-mouse IgG H&L			1:5,000/WB	HRP	CW Biotech (CW0102S)
Goat anti-rabbit IgG H&L			1:5,000/WB	HRP	ABclonal(AS014)
Donkey anti-rabbit IgG H&L			1:1,000/IF	Alexa Fluor^®^ 647	Abcam(ab150075)
Donkey anti-goat IgG H&L			1:500/IF	Alexa Fluor^®^ 555	Abcam(ab15013)
Donkey anti-mouse IgG H&L			1:1,000/IF	Alexa Fluor^®^ 488	Abcam(ab150105)

WB, western blot; IF, immunofluorescence; M, monoclonal; P, polyclonal.

### Animals and Stereotactic Injections

Male ICR mice (25–30 g, 6 w) were purchased from Vital River Experimental Animal CO. LTD (Beijing, China). Animals were housed with free access to standard food and water under a 12 h light/dark cycle with controlled room temperature and humidity. Animals were habituated for 1 week to the new environment before initiating surgery. All procedures were conducted in accordance with the protocols approved by the Animal Ethical Committee at the Institute of Medicinal Plant Development, Chinese Academy of Medical Sciences.

Mice were anesthetized with 2% isoflurane and placed in a stereotaxic apparatus (Benchmark, USA). The Aβ_1–42_ oligomers preparations (50 μM) or an equivalent volume of vehicle (1% DMSO in PBS) were injected into the bilateral CA1 molecular layer of hippocampus (1 μL in each side) using a Hamilton syringe (10 μL, 701N, ga26s/51 mm/pst3; Hamilton, Bonaduz, GR, Switzerland) and mounted on a nano-pump syringe (Benchmark, USA) with a speed of 0.4 μL/min. The coordinates of the injection sites were adapted from the Paxinos and Franklin atlas (Paxinos and Franklin, 2001): −2.0 mm posterior to bregma, ± 1.5 mm lateral to the midline, and 1.5 mm deep from dura. After injection, the needle was left in place for another 5 min and withdrawn slowly.

### Groups and Drug Administration

**Figure 1:**
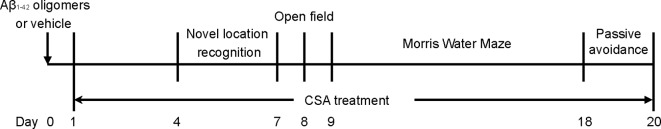
Experimental schedule of behavioral performance follow-up.

The mice were numbered according to their body weight and then randomly divided into six experimental groups using a random number table: (1) a control group (without stereotactic injections); (2) a sham-operated group; (3) Aβ_1–42_ oligomers group; (4) Aβ_1–42_ oligomers + CSA 7.5 mg/kg/d group; (5) Aβ_1–42_ oligomers + CSA 15 mg/kg/d; (6) Aβ_1–42_ oligomers + CSA 30 mg/kg/d. CSA (98.0% purity by HPLC) was separated from the leaves of Pigeon pea in our laboratory (Liu et al., 2014b), suspended in 0.5% carboxymethylcellulose sodium (CMC-Na) water solution and intragastrically administered after surgery. Meanwhile, the control, sham and Aβ_1–42_ oligomers groups were received the same volume of 0.5% CMC-Na water solution intragastrically. Learning and memory capacity was assessed using object location recognition (OLR), open filed, MWM, and passive avoidance tests. The experimental schedule is shown in **Figure 1**. Another same experiment was operated without behavioral tests, and mice were sacrificed for tissue collection seven days after stereotactic injections.

### Behavioral Tests

#### Object Location Recognition (OLR) Test

Object location memory was measured using published methods (Dong et al., 2019). The test was performed in an open field arena, to which animals were first habituated for three consecutive days. The next day, two identical objects were introduced to the arena. In familiar trials, mice were allowed to freely explore the arena with the two objects for 5 min. In test trials 20 min thereafter, mice again encountered the two objects for 5 min, except that one of them had been moved to a new location. The trials were recorded using a camera mounted above the arena and scored for the amount of time spent sniffing or touching the objects. The object-location discrimination index is calculated as dividing the time spent exploring the object in the novel location over the total exploring time.

#### Open Field Test

The locomotor activity of mice was assessed using an open-field computer-aided control system (Lu et al., 2018). After the OLR test, mice were placed in a square box and allowed to freely move for 10 min while being recorded by an overhead camera. The total distance of moving was recorded automatically.

#### Morris Water Maze (MWM) Test

The spatial memory was evaluated according to the protocol of MWM (Vorhees and Williams, 2006). A black stainless-steel circular tank (100 cm diameter, 40 cm height) was filled with water (23 ± 1°C) being opaque by adding nontoxic black ink and divided into four equal quadrants. A black circular escape platform (6 cm diameter) was submerged 1.5 cm below the water surface. Some visual cues surrounding this tank kept permanent place throughout all MWM tests. Mice were monitored by a video camera tracking system directly above the water tank as they swim. Parameters were measured using the software system developed by the China Astronaut Center and the Institute of Medicinal Plant Development, Chinese Academy of Medical Sciences, and Peking Union Medical College.

In the spatial acquisition test, mice were trained three trials per day for five consecutive days. The platform was placed in the target quadrant, and one of the other three quadrants was chosen in each trial as the start location in a semi-random set. Before each trial, mice were allowed to stay in the platform for 10 s. After that, mice were released into the water and were given a maximum of 90 s to find the platform. If mice did not find the platform within 90 s, they were guided to the platform and assigned an escape latency of 90 s for this trial. Once the mice reached the platform, they were allowed to stay on the platform for 10 s and then dried and returned to their home cage.

Twenty-four hours after the last training trial, a probe test was performed in which the platform was removed. The start location was opposite to the target quadrant and each mouse completed a single, 90-s probe test. The number of platform crossings was recorded to assess reference memory.

After the probe test, the working memory test was performed in which the platform was relocated every day for three consecutive days (Zhou et al., 2019). Mice were given two trials per day. In the first trial (acquisition), mice had to learn the new location of the platform by trial-and-error in 90 s before staying in the platform for 15 s. After the 15-s inter-trial interval, the second trial (retrieval) was performed. Mice started from a different point with the same platform position. If the mouse recalled the first trial, it would swim to the goal faster on the second trial. As the platform was moved daily, no learning of platform position from the previous day could be transferred to the next day’s task. Therefore, recall on each day during the second trial was dependent on that day’s first trial and measured only temporary or working memory. The average escape latency of each trial for three days was recorded to assess working or trial-dependent learning and memory.

#### Passive Avoidance Task

The passive avoidance test was carried out in a trough-shaped apparatus consisted of a white illuminated chamber and a dark chamber (20 cm × 12 cm × 60 cm, respectively) as previously reported (Xue et al., 2010; Bu et al., 2011; Reid et al., 2018). On the first day, mice were allowed to explore both chambers for 4 min twice to acclimatize. Next day the training trial was performed for 5 min after a 3-min adaption. When mice entered the dark chamber, an electric foot shock (40 V, 2 mA) was delivered. After 24 h, the consolidation trial was performed for 5 min in the same way as training and the tracking system was started once mice were placed into the light chamber. The step-through latency (up to a maximum of 300 s) and error times to enter the dark chamber were recorded.

### Nissl Staining and Immunofluorescence

Three mice in each group were perfused transcardially with PBS, followed by cold 4% paraformaldehyde (PFA) in PBS. Brains were removed and postfixed in 4% PFA in PBS at 4ºC overnight and then immersed in a freshly made 15% sucrose solution in PBS. Next day, brains were transferred to 30% sucrose solution in PBS for cryoprotection. After 24 h, brains were embedded in optimal cutting temperature compound (Sakura Finetek USA, Inc., Torrance, CA, USA) and frozen quickly onto specimen discs with quick-freeze shelf in the freezing microtome (Leica CM3050 S, Nussloch, Germany). Serial 10 μm-thick coronal sections were cut and thaw mounted on positively charge adhesion slides which were stored at −20°C until use.

Nissl staining was carried out as described before (Lindroos and Leinonen, 1983). Brain sections were dehydrated in ascending grades of ethanol, immersed in xylene, rehydrated in descending grades of ethanol, and hydrated in distilled water. Subsequently, the sections were stained with toluidine blue, dehydrated in ethanol and cleared in xylene before coverslipping.

For immunofluorescence analysis, brain sections were briefly rinsed in PBS three times for 5 min and incubated in PBS containing 0.2% Triton X-100 for 30 min. After rinsing in PBS three times, the sections were incubated with 2% BSA in PBST (0.2% Tween 20 in PBS) for 1 h to block unspecific binding of the antibodies. Primary antibodies (**Table 1**) were diluted in blocking solution and incubated with sections overnight at 4°C, followed by incubation with Alexa Fluor 488, Alexa Fluor 555, and Alexa Fluor 647-conjugated secondary antibodies (**Table 1**) for 1 h at room temperature in the dark. After rinsing in PBS three times, coverslips were mounted with mounting medium containing the nuclear counterstain DAPI (4’,6-diamidino-2-phenylindole, SouthernBiotech) and sealed with nail polish to prevent drying and movement.

### Image Analysis

All sections were digitalized at 20× magnification utilizing a TissueFaxs System coupled with a Zeiss Axio Imager Z2 Microscope (Jena, Germany). Hippocampal regions were acquired using the TissueFaxs (TissueGnostics, Vienna, Austria) under the same exposure time and acquisition parameters. With a threshold that best discriminated staining from the background, the integrated optical density (IOD, for Nissl or Aβ analysis) and area (for GFAP or Iba1 analysis) above the threshold were calculated for each image with equal size by ImageJ software (NIH version). For each hippocampal region (CA1, CA3, and DG), the obtained value was first normalized with respect to control, and the values from the different levels were then averaged.

### Hippocampus Levels of Glu and GABA

Glu and GABA levels in mouse hippocampus were measured by a liquid chromatography-tandem mass spectrometry (LC-MS/MS) analysis method as published before (Wang et al., 2019). Briefly, mice were sacrificed by decapitation and hippocampus was rapidly separated, weighed and homogenized with water in an ice bath. An aliquot of 50 μL hippocampus homogenate was spiked with 20 μL internal standard solution (500 ng/mL), followed by mixing with 10 μL trifluoroacetic acid to precipitate protein. The mixture was vortexed and centrifuged at 4°C, 20,000 × g for 15 min. The supernatant was collected and a 1 µL aliquot was injected into the LC-MS/MS system. Gradient elution was performed on a Restek Ultra Aqueous C18 column (100 mm × 2.1 mm, 3 µm, Bellefonte, PA, USA) using acetonitrile and water containing 0.1% FA as the mobile phase. The mass detection was performed by conducting multiple reaction monitoring (MRM) analysis with an electrospray ionization source in positive mode. The MRM transitions are as follows: *m/z* 148.2→84.0 (Glu), 104.1→87.0 (GABA), and 152.0→109.9 (4-acetamidophenol, internal standard).

### Western Blot

Total proteins from hippocampus tissues were using lysis buffer containing 1% protease inhibitor cocktail and 1% phosphatase inhibitor cocktail (CW Biotech, Beijing, China). Protein concentrations were determined by BCA assay. Protein samples were separated by SDS-PAGE and transferred to nitrocellulose membranes. The membranes were blocked with 7.5% nonfat dried milk for 2 h and incubated with primary antibodies (**Table 1**) at 4°C overnight. Next day, membranes were subsequently washed with TBST (0.05% Tween 20 in Tris-buffered saline) three times and incubated with the appropriate horseradish peroxidase-conjugated secondary antibody (**Table 1**) at room temperature for 2 h. After washing three times with TBST, the protein bands were visualized using an ECL system (CW Biotech, Beijing, China), imaged in a Bio-Rad imaging system (Bio-Rad, Hercules, CA, USA) and analyzed by ImageJ.

### Statistical Analysis

Data were analyzed with SPSS version 16.0 and expressed as mean ± SEM. The escape latency during spatial acquisition in the MWM test was examined by repeated measures analysis of variance (ANOVA) followed by Fisher’s least significant difference (LSD) *post hoc* test. Other data were analyzed using one-way ANOVA followed by LSD *post hoc* test (only for normality parameters with equal variances) or using the Mann-Whitney *U* test. Statistical significance was considered when *p* < 0.05.

## Results

### Aβ_1–42_ Oligomers Characterization

**Figure 2:**
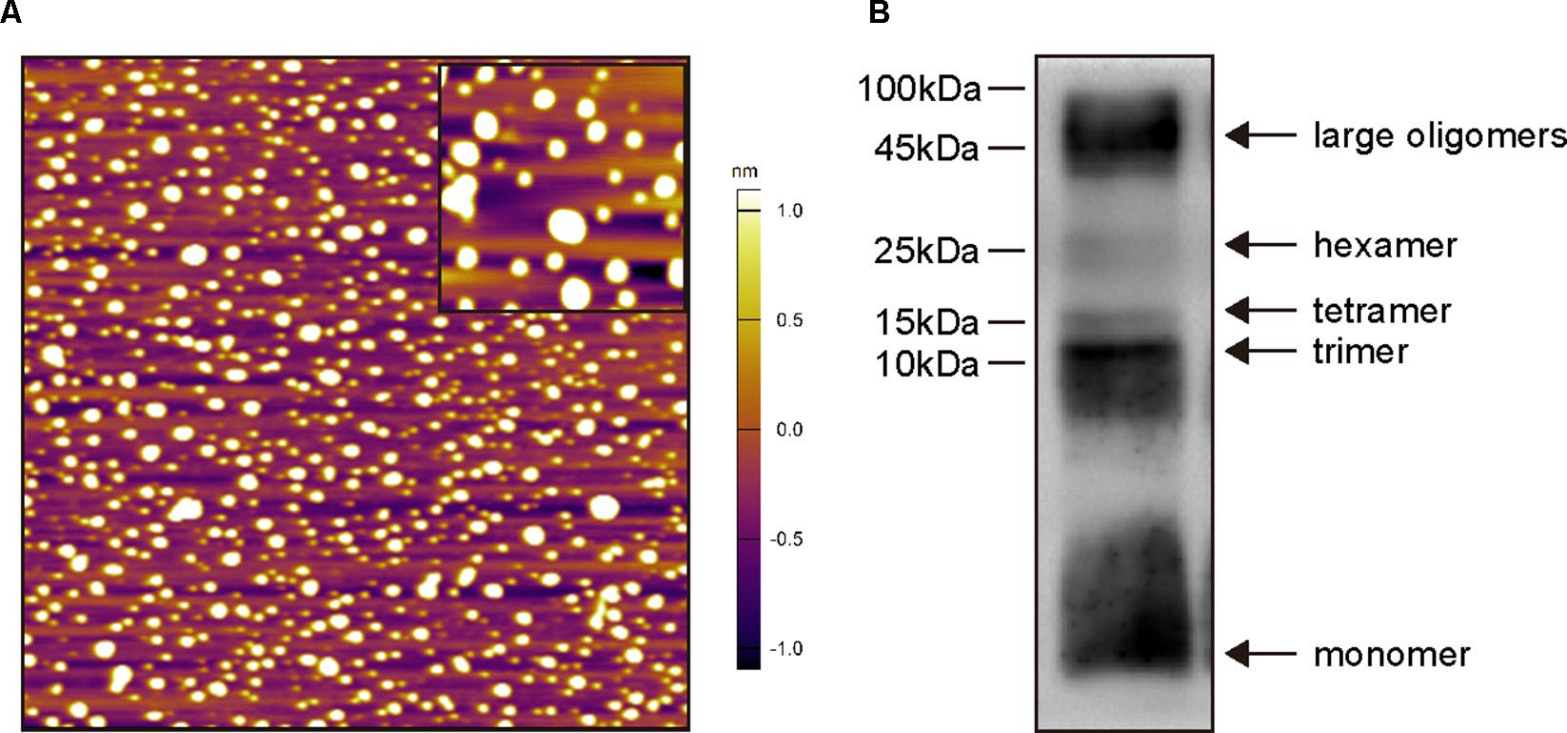
Biophysical characterization of Aβ_1–42_ oligomers preparations. **(A)** Atomic force microscopy images of Aβ_1–42_ oligomers preparations. Only the globular structure of oligomers was observed in the representative 2 × 2-μm x–y image (inset image, 350 × 350-nm x–y). **(B)** Western blot analysis of the Aβ_1–42_ oligomers preparations which were separated by SDS-PAGE and probed with monoclonal antibody 6E10 (recognizing residues 1–16 of Aβ). Monomers, trimers, tetramers, hexamers and large oligomers are visible.

Before *in vivo* experiments, oligomeric Aβ_1–42_ structures were characterized by AFM (**Figure 2A**) and Western blot analysis (**Figure 2B**). As shown in **Figure 2A**, Aβ_1–42_ assembled into predominantly 1–6-nm globular structures after incubation, and no fibril formation was detected under this condition. Western blot analysis of SDS-PAGE (**Figure 2B**) revealed the preparations contained a mixture of Aβ_1–42_ monomers, trimers, and tetramers, hexamers and large oligomers. Taken together, these data indicate that the predominant compositions of the present soluble Aβ_1–42_ preparations were Aβ_1–42_ monomers and oligomers rather than fibrils.

### CSA Treatment Rescued Aβ_1–42_ Oligomers-Induced Impairment of Mice Learning and Memory

**Figure 3:**
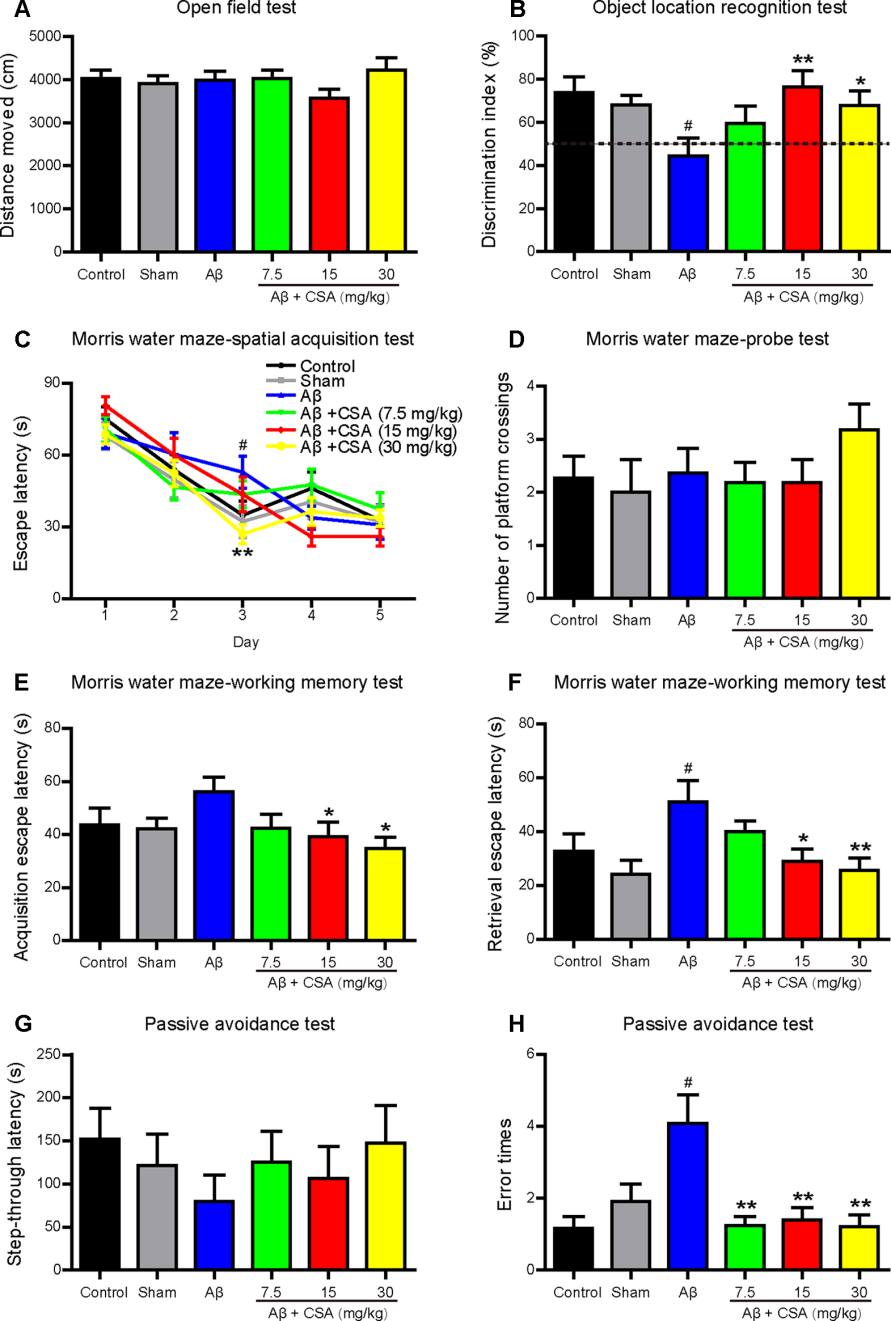
Effects of CSA on cognitive impairment of mice induced by intrahippocampal injection of Aβ_1–42_ oligomers. **(A)** Distance traveled in the open field test during 10 min (Mann-Whitney *U* test, n = 11–12 mice per group). **(B)** The discrimination index in test trials of object location recognition test (one-way ANOVA followed by LSD *post hoc* test, n = 10–12 mice per group). **(C)** The mean daily escape latency calculated from three trials per day in spatial acquisition test of Morris water maze (MWM) (repeated measures ANOVA followed by LSD *post hoc* test, n = 11 mice per group). **(D)** The number of times that the mice crossed the original platform location in the probe test of MWM (Mann-Whitney *U* test, n = 11 mice per group). Acquisition **(E)** and retrieval **(F)** escape latency in working memory test of MWM (Mann-Whitney *U* test, n = 9–11 mice per group). The step-through latency **(G)** and error times **(H)** into the dark chamber in the consolidation trial of passive avoidance test (Mann-Whitney *U* test, n = 9–12 mice per group). All values are mean ± SEM. ^#^ *P* < 0.05 as compared to the sham-operated group. *P < 0.05 and **P < 0.01 as compared to the Aβ_1–42_ oligomers-injected group.

Open field test was used to determine the spontaneous motor activity. Mice traveled a similar distance without significant changes among all groups (**Figure 3A**). The results indicate that CSA had no effects on the locomotor activity of mice.

To investigate the effects of CSA on cognitive performance, mice were tested in OLR, MWM, and passive avoidance tests. The OLR test is based on the spontaneous tendency for rodents to spend more time exploring a novel location rather than a familiar location to assess spatial memory and discrimination. Mice showed no preference for the two locations during familiar trials. In test trials (**Figure 3B**), like in control group, mice in the sham-operated group spent more time for exploring the novel location than the familiar location with a discrimination index nearly 70%. In contrast, the discrimination index of Aβ_1–42_ oligomers-injected group was decreased significantly to around 50% compared with that of the sham-operated group. Treatments with CSA (15 and 30 mg/kg) elevated the discrimination index with significance (*P* = 0.003, *P* = 0.026), suggesting that CSA (15 and 30 mg/kg) treatments could reverse Aβ_1–42_ oligomers-induced cognitive deficits.

In spatial acquisition test of MWM, the escape latency gradually decreased during the first three training days in all groups (**Figure 3C**). The repeated measurement of ANOVA showed the interaction effect between group and day on escape latency was significant (*P* = 0.018), indicating that the effect of group varied with day. Then the group effect on each day was measured by LSD *post hoc* test to localize which day showed group differences. Longer escape latency was observed in Aβ_1–42_ oligomers-injected group on day 3 in contrast to that in the sham-operated group (*P* = 0.020), indicating that the spatial learning ability of mice was significantly affected by Aβ_1–42_ oligomers. On the same training day, CSA (30 mg/kg) treatment significantly shortened the escape latency prolongation caused by Aβ_1–42_ oligomers (*P* = 0.004). In probe test, the number of platform crossings of mice in CSA treatment (30 mg/kg) group was the highest among all groups even without statistical significance compared with that in Aβ_1–42_ oligomers-injected group (**Figure 3D**). These results suggest that intrahippocampal injection of Aβ_1–42_ oligomers impaired mice spatial learning and memory and CSA treatment rescued the impairment. In working memory test, the acquisition escape latency was decreased in Aβ_1–42_ oligomers-injected group (*P* = 0.060) while CSA (15 and 30 mg/kg) treatments reversed the decrease significantly (*P* = 0.034, *P* = 0.011, **Figure 3E**). In the retrieval trial, the latency was significantly decreased in Aβ_1–42_ oligomers-injected group (*P* = 0.018), and CSA (15 and 30 mg/kg) treatments reversed the decrease significantly (*P* = 0.022, *P* = 0.007, **Figure 3F**) as well. These findings indicate that CSA treatment could enhance both acquisition and retrieval of Aβ_1–42_ oligomers-impaired working memory.

The passive avoidance task is a fear-aggravated test used to evaluate non-spatial learning and memory. In the consolidation trial (**Figures 3G, H**), Aβ_1–42_ oligomers-induced cognitive deficits were observed as shorter latency and more error times (*P* = 0.035) into the dark chamber than those of sham-operated mice. Compared with Aβ_1–42_ oligomers-injected group, CSA (7.5, 15, and 30 mg/kg) treatments reversed the increase of error times significantly (*P* = 0.002, *P* = 0.009, *P* = 0.005), and prolonged the latency even without statistical significance.

### Aβ_1–42_ Oligomers Injection and CSA Treatment Did Not Cause Hippocampal Neuronal Loss

**Figure 4:**
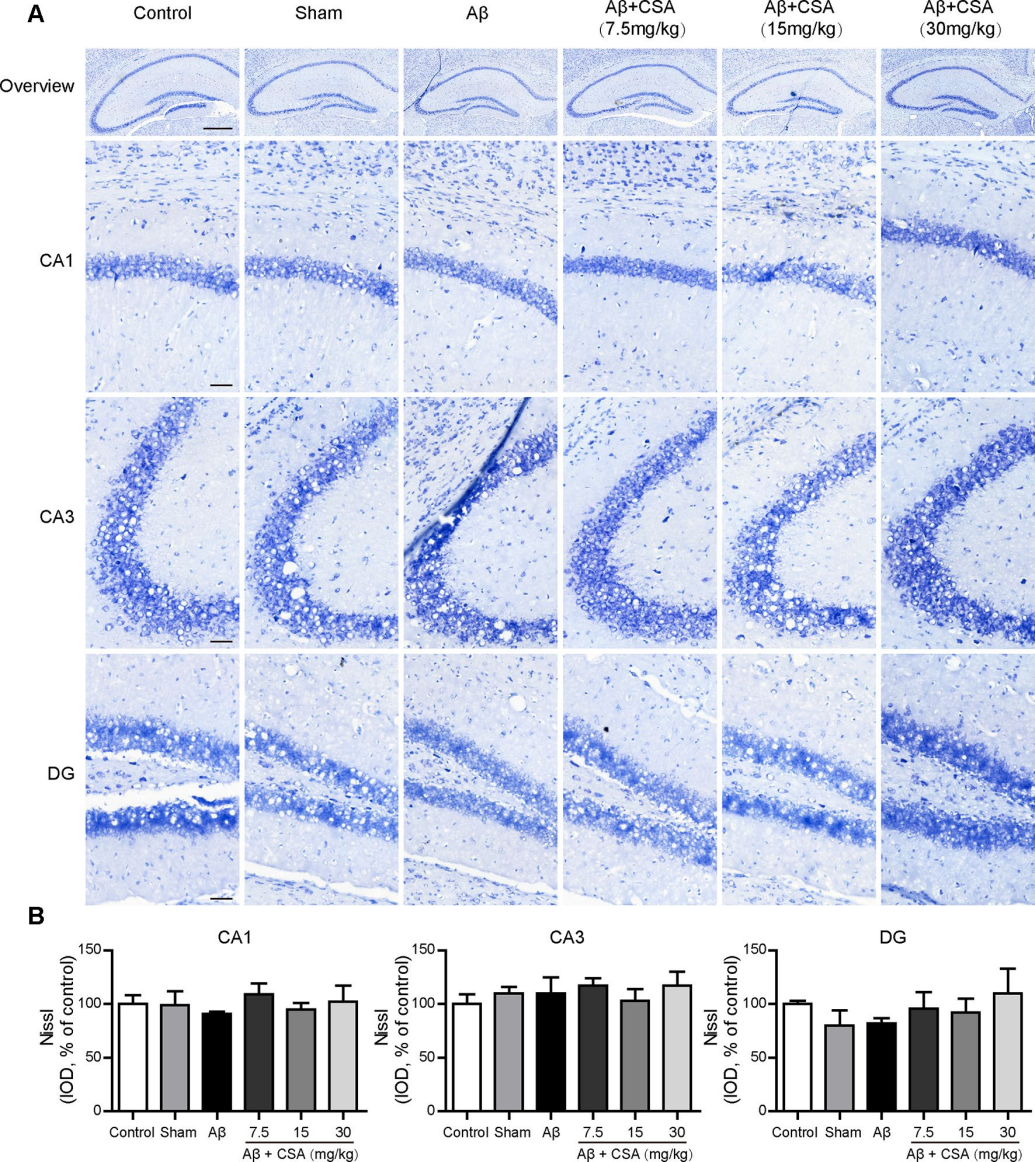
Effects of CSA on the status of the hippocampal neuron of mice induced by intrahippocampal injection of Aβ_1–42_ oligomers. **(A)** Representative micrographs of Nissl staining in the hippocampus. The scale bar represents 500 μm in the overview image of hippocampus or 50 μm in the detail image of hippocampal subregions (CA1, CA3, and DG). **(B)** Quantification of Nissl integrated optical density (IOD) in the CA1, CA3, and DG regions of the hippocampus (one-way ANOVA followed by LSD *post hoc* test, n = 3 mice per group). Values represent mean ± SEM.

The protective effects of CSA were investigated using Nissl staining in mice hippocampus. Neurons from all groups had a distinct profile and a spherical nucleus neatly arranged with no difference in neuronal morphology (**Figure 4A**). The IOD of Nissl bodies was quantified to show neuronal status. It was found that there were no significant differences in the hippocampal CA1, CA2, or DG cell layers between control and any other groups (**Figure 4B**). Therefore, no detectable hippocampal neuronal loss was observed after the injection of Aβ_1–42_ oligomers and CSA treatment, suggesting that the protective effects of CSA did not involve in the modulation of neuron death.

### CSA Treatment Attenuated Aβ_1–42_ Oligomers-Induced Activation of Microglia and Reactivity of Astrocytes in the Hippocampus

**Figure 5:**
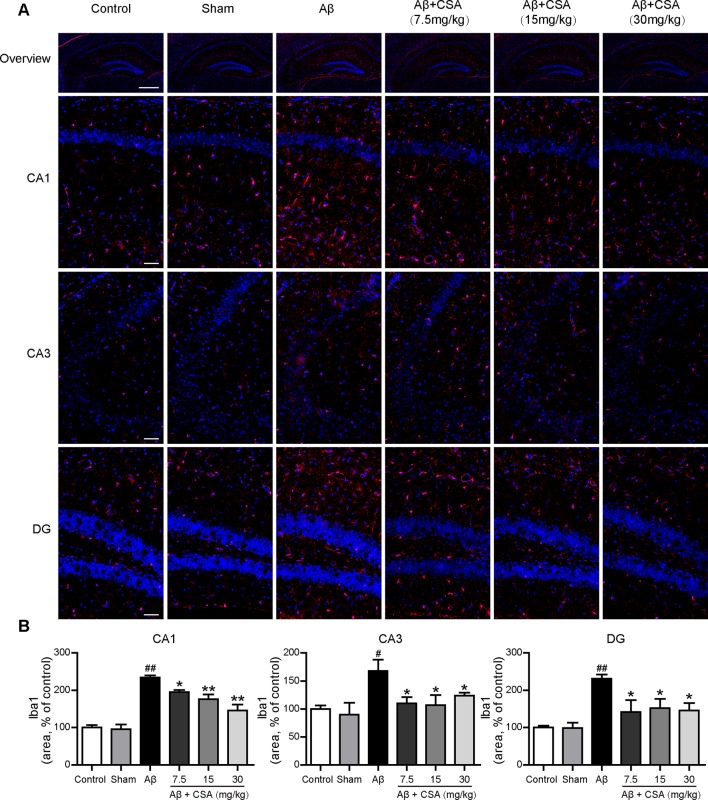
Effects of CSA on microglial activation in the hippocampus of mice induced by intrahippocampal injection of Aβ_1–42_ oligomers. **(A)** Representative micrographs showing Iba1 (red) immunofluorescence in the hippocampus. Nuclei are in blue (DAPI). The scale bar represents 500 μm in the overview image of hippocampus or 50 μm in the detail image of hippocampal subregions (CA1, CA3, and DG). **(B)** Quantification of Iba1 area in the CA1, CA3 and DG regions of the hippocampus (one-way ANOVA followed by LSD *post hoc* test or Mann-Whitney *U* test, n = 3 mice per group). Values represent mean ± SEM. ^#^ *P* < 0.05 and ^##^ *P* < 0.01 as compared to the sham-operated group. *P < 0.05 and **P < 0.01 as compared to the Aβ_1–42_ oligomers-injected group.

**Figure 6:**
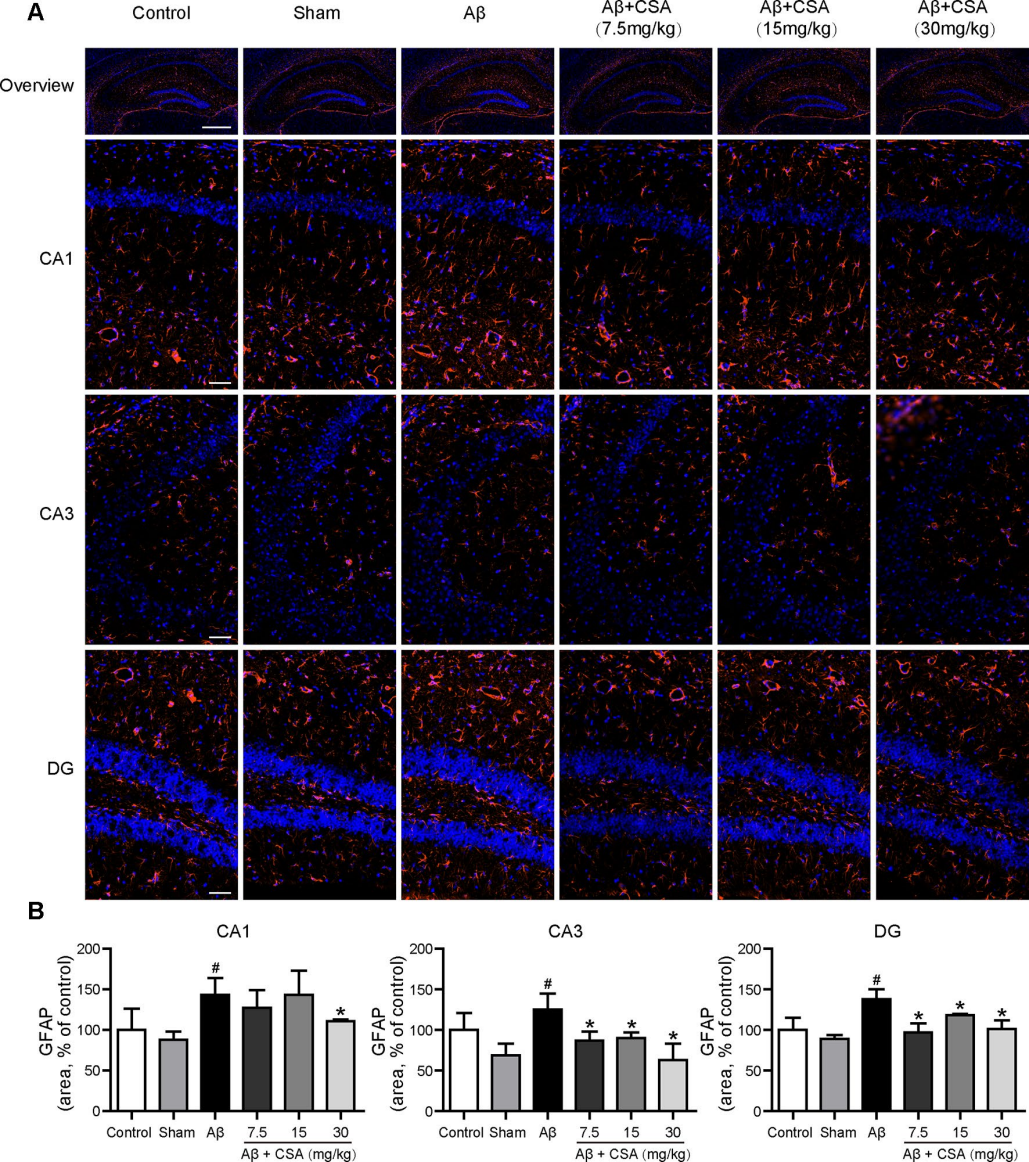
Effects of CSA on astrocyte reactivity in the hippocampus of mice induced by intrahippocampal injection of Aβ_1–42_ oligomers. **(A)** Representative micrographs showing GFAP (orange) immunofluorescence in the hippocampus. Nuclei are in blue (DAPI). The scale bar represents 500 μm in the overview image of hippocampus or 50 μm in the detail image of hippocampal subregions (CA1, CA3, and DG). **(B)** Quantification of GFAP area in the CA1, CA3, and DG regions of the hippocampus (Mann-Whitney *U* test, n = 3 mice per group). Values represent mean ± SEM. ^#^ *P* < 0.05 as compared to the sham-operated group. *P < 0.05 as compared to the Aβ_1–42_ oligomers-injected group.

Sections from the hippocampus were further immunostained for the presence of microglia (anti-Iba1 antibody) and astrocytes (anti-GFAP antibody). Compared with the sham-operated group, Aβ_1–42_ oligomers-injected group showed markedly increased immunoreactivities for both Iba1 and GFAP in the hippocampus (**Figures 5** and **6**). The activation of microglia and reactivity of astrocytes by Aβ_1–42_ oligomers were attenuated by CSA treatment significantly (**Figures 5** and **6**).

### CSA Treatment Reduced Aβ_1–42_ Levels in the Hippocampus

**Figure 7:**
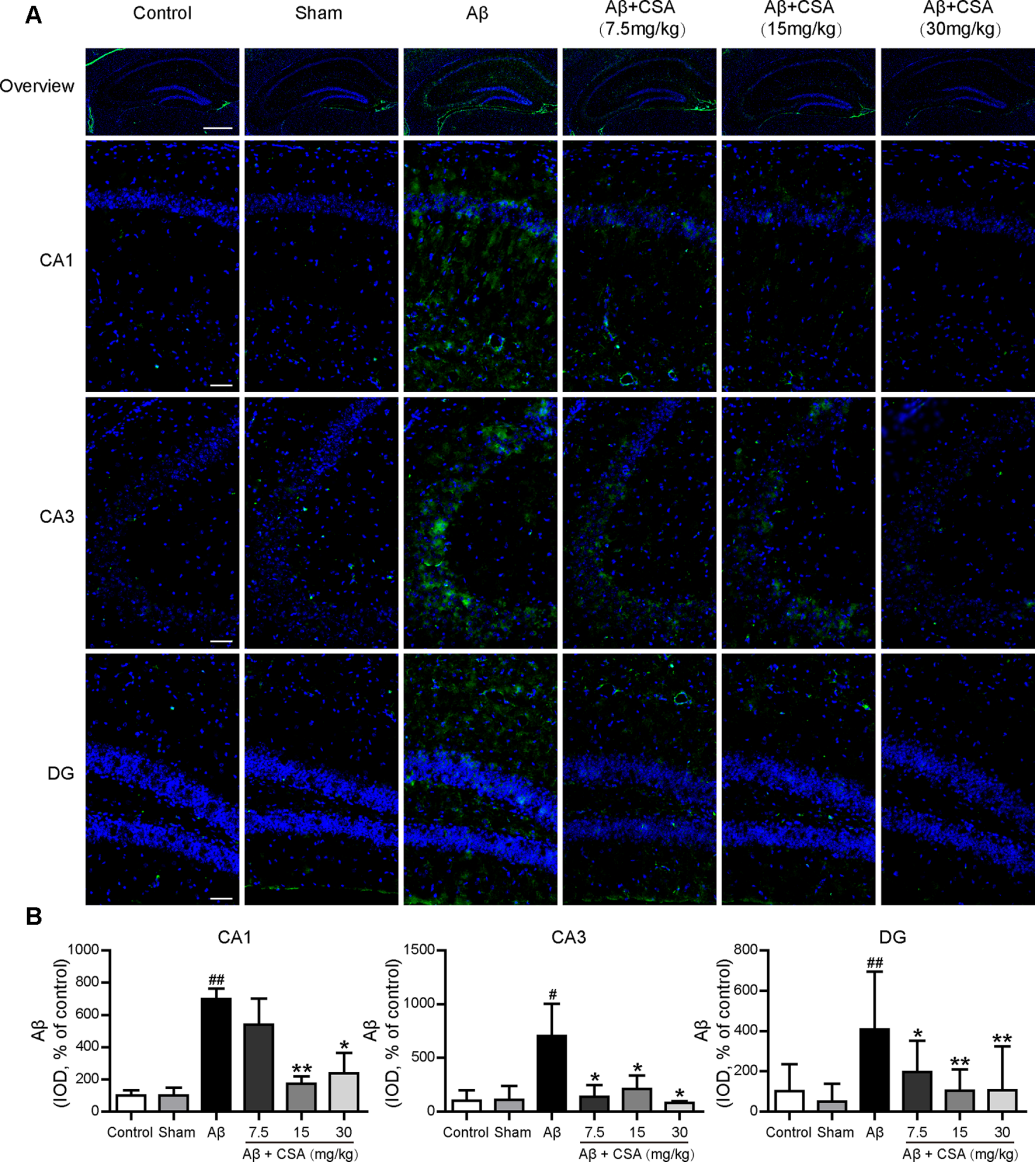
Effects of CSA on hippocampal Aβ_1–42_ levels of mice induced by intrahippocampal injection of Aβ_1–42_ oligomers. **(A)** Representative micrographs showing Aβ (green) immunofluorescence in the hippocampus. Nuclei are in blue (DAPI). The scale bar represents 500 μm in the overview image of hippocampus or 50 μm in the detail image of hippocampal subregions (CA1, CA3, and DG). **(B)** Quantification of Aβ integrated optical density (IOD) in the CA1, CA3, and DG regions of the hippocampus (one-way ANOVA followed by LSD *post hoc* test or Mann-Whitney *U* test, n = 3 mice per group). Values represent mean ± SEM. ^#^ *P* < 0.05 and ^##^ *P* < 0.01 as compared to the sham-operated group. *P < 0.05 and **P < 0.01 as compared to the Aβ_1–42_ oligomers-injected group.

Immunostaining with anti-Aβ (6E10) confirmed the widespread diffusion of Aβ oligomers after intracerebral injection. All hippocampal regions (CA1, CA3, and DG) of Aβ_1–42_ oligomers-injected mice presented significantly abundant Aβ immunoreactivity compared with those of sham-operated mice (*P* = 0.004, *P* = 0.049, *P* = 0.002). In the CA1 region, CSA (15 and 30 mg/kg) treatments, except the lowest dose (7.5 mg/kg), reduced Aβ_1–42_ levels significantly compared with Aβ_1–42_ oligomers-injected group (*P* = 0.009, *P* = 0.020). In the CA3 and DG regions, all doses of CSA (7.5, 15 and 30 mg/kg) could reduce Aβ_1–42_ levels significantly (**Figure 7**).

### CSA Treatment Reversed Aβ_1–42_ Oligomers-Induced Dyshomeostasis of Glu and GABA in the Hippocampus

**Figure 8:**
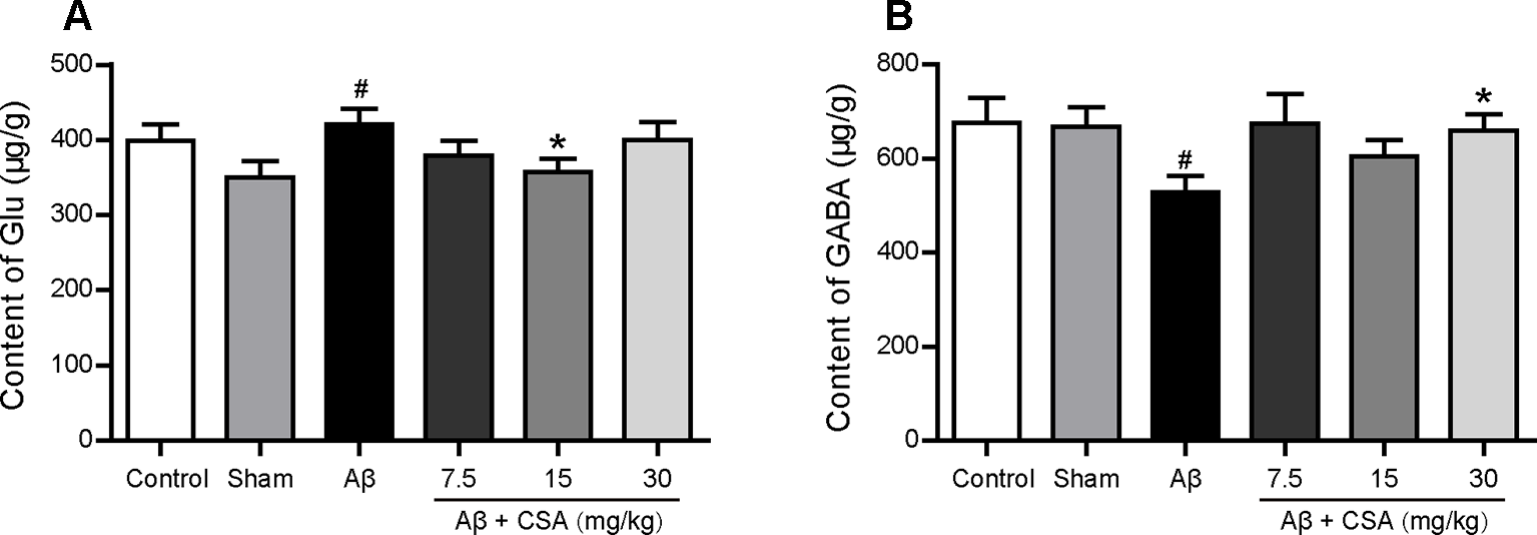
Effects of CSA on hippocampal dyshomeostasis of glutamate (Glu) and γ-aminobutyric acid (GABA) of mice induced by intrahippocampal injection of Aβ_1–42_ oligomers. **(A)** The content of Glu in the hippocampus (Mann-Whitney *U* test, n = 7–9 mice per group). **(B)** The content of GABA in the hippocampus (Mann-Whitney *U* test, n = 7-9 mice per group). All values are mean ± SEM. ^#^ *P* < 0.05 as compared to the sham-operated group. ^*^ *P* < 0.05 as compared to the Aβ_1–42_ oligomers-injected group

The concentrations of Glu and GABA in the hippocampus are shown in **Figure 8**. Compared with the sham-operated group, Glu levels were significantly increased and GABA levels were significantly decreased in the hippocampus of Aβ_1–42_ oligomers-injected group (*P* = 0.035, *P* = 0.049). CSA treatment significantly decreased Glu levels at the dose of 15 mg/kg (*P* = 0.035) and significantly increased GABA levels at the dose of 30 mg/kg (*P* = 0.037) compared with Aβ_1–42_ oligomers-injected group.

### CSA Treatment Prevented Aβ_1–42_ Oligomers-Induced Increase of GluN2B and Decrease of Downstream PKA/CREB/BDNF/TrkB Signaling Pathway

**Figure 9:**
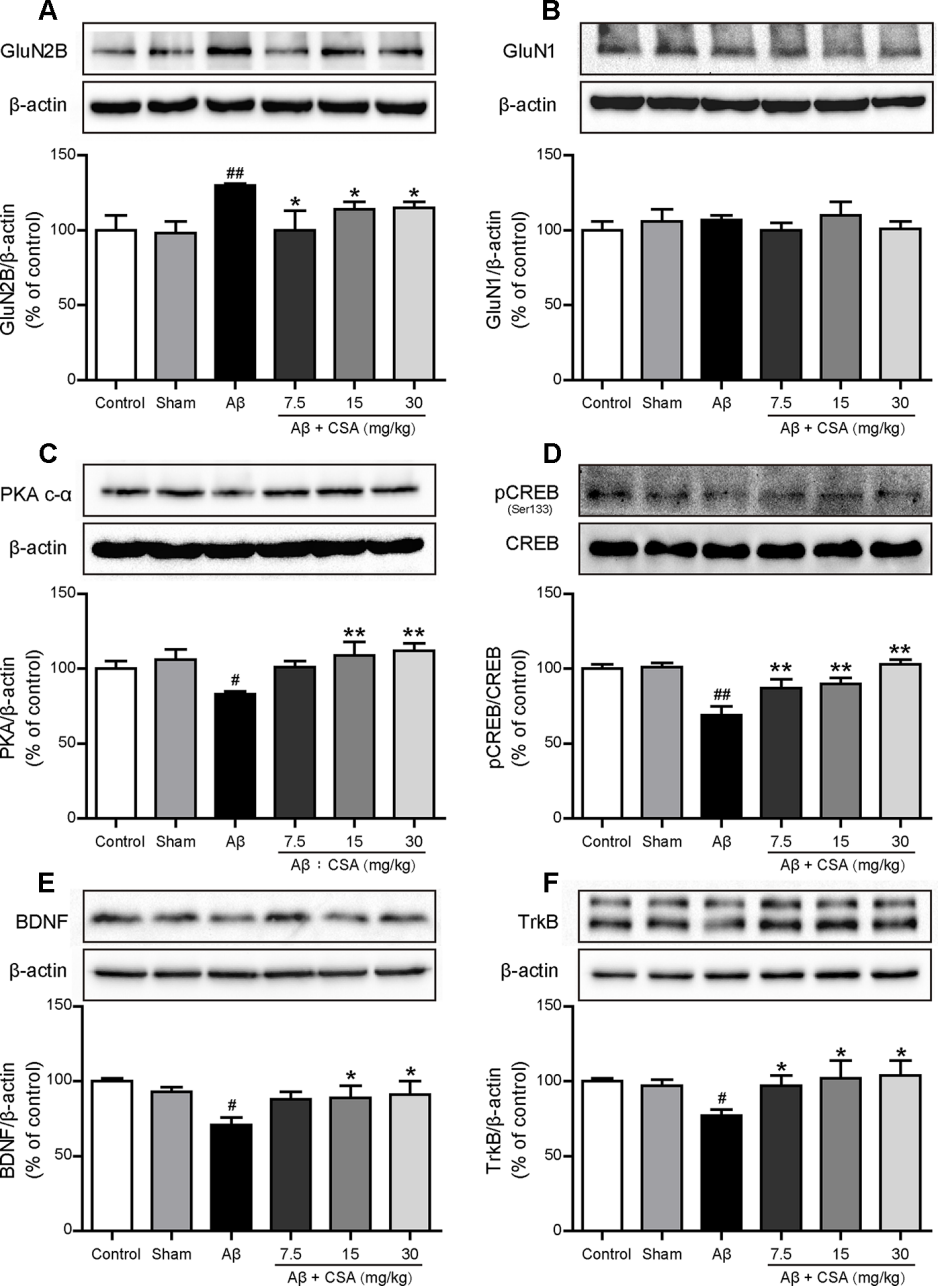
Effects of CSA on hippocampal expression of GluN2B, GluN1 and PKA/CREB/BDNF/TrkB signaling pathway of mice induced by intrahippocampal injection of Aβ_1–42_ oligomers. **(A)** The expression levels of GluN2B normalized to β-actin (Mann-Whitney *U* test, n = 6 mice per group). **(B)** The expression levels of GluN1 normalized to β-actin (one-way ANOVA followed by LSD *post hoc* test, n = 7 mice per group). **(C)** The expression levels of PKA normalized to β-actin (one-way ANOVA followed by LSD *post hoc* test, n = 5 mice per group). **(D)** The ratio of pCREB/CREB expression levels normalized to β-actin (one-way ANOVA followed by LSD *post hoc* test, n = 5 mice per group). **(E)** The expression levels of BDNF normalized to β-actin (one-way ANOVA followed by LSD *post hoc* test, n = 5 mice per group). **(F)** The expression levels of TrkB normalized to β-actin (Mann-Whitney *U* test, n = 5 mice per group). Values represent mean ± SEM. ^#^ *P* < 0.05 and ^##^ *P* < 0.01 as compared to the sham-operated group. *P < 0.05 and **P < 0.01 as compared to the Aβ_1–42_ oligomers-injected group.

To investigate the protective effects of CSA, Western blot analysis was used to measure the expression levels of GluN2B, GluN1, PKA c-α, pCREB/CREB, BDNF, and TrkB. As shown in **Figure 9A**, GluN2B expression was markedly increased after Aβ_1–42_ oligomers injection (*P* = 0.004) but reduced significantly when treated with CSA (*P* = 0.035, *P* = 0.045, *P* = 0.013). However, there was no significant difference of GluN1 levels among all groups (**Figure 9B**). In the subsequent signaling pathway, the expression of PKA c-α, pCREB/CREB, BDNF or TrkB was downregulated in Aβ_1–42_ oligomers-injected group compared with those in the sham-operated group (*P* = 0.016, *P* = 0.00002, *P* = 0.016, and *P* = 0.016) but upregulated by CSA treatments (**Figures 9C–F**).

## Discussion

The Aβ injection model has been used widely, but there are still missing operation standards. Numerous factors can affect the outcome of this model, such as the preparation methods of Aβ, injection doses, injection sites, and the duration of modeling. Therefore, we validated these factors before using this model to evaluate the effects of CSA on learning and memory. Distinct Aβ aggregates are formed by depending on the initial structure in lyophilized stocks of commercial Aβ, Aβ species and the incubation conditions, including concentration, time, temperature, pH, and ionic strength. Many studies (Choi et al., 2012; Yang et al., 2012; Choi et al., 2013; Chen et al., 2015) adopted the incubation method of Aβ peptides in Maurice’s report (Maurice et al., 1996). Aβ peptides were directly dissolved in sterile double-distilled water (or saline or PBS) and incubated at 37 ºC for 4 days. Light microscopic observation indicated that two types of insoluble precipitates birefringent fibril-like structures and globular aggregates were formed after incubation (Maurice et al., 1996). Different preexisting structures in lyophilized stocks of commercial Aβ lead to different types of Aβ aggregates after the same incubation condition, which might result in distinct outcomes of *in vivo* or *in vitro* experiments. Therefore, the removal of any preexisting structures in lyophilized stocks of Aβ peptide by HFIP is required for controlling aggregation studies (Stine et al., 2003). After a standard incubation method, we characterized the structures of Aβ_1–42_ aggregates by AFM and Western blot analysis. The results indicate that the predominant compositions of the present soluble Aβ_1–42_ preparations were Aβ_1–42_ monomers and oligomers rather than fibrils (**Figure 2**). The clarified structures of Aβ_1–42_ preparations laid a critical foundation for exploring the *in vivo* mechanism of soluble Aβ_1–42_ oligomers neurotoxicity and CSA treatment.

The amount of injected Aβ was calculated to reach a concentration of the same order of magnitude as the actual level of soluble Aβ observed in AD brains. The mean concentration of soluble Aβ in the brains of AD patients was 0.29 μg/g in the study of McLean et al. (1999) or 1.5 μg/g in the study of Tabaton and Piccini (2005), while that of normal subjects was at a quite low level (ng/g). The amount of intrahippocampal injection of Aβ_1–42_ oligomers in the study of Epelbaum et al. (2015) was 50 pmol/mouse which could induce memory impairment. Other amounts, such as 10, 30, 222, and 500 pmol/mouse, were used as well (Youssef et al., 2008; Dineley et al., 2010; Choi et al., 2011; Brito-Moreira et al., 2017). After taking these references into account, we injected an aliquot of 2 μL Aβ_1–42_ oligomers preparations (50 μM), equivalent to 100 pmol of Aβ_1–42_ monomer, into the mouse brain (about 500 mg). If the Aβ_1–42_ preparations freely diffused in the whole brain, the final concentration would be 0.9 μg/g, reaching to a similar concentration in the brains of AD patients. Therefore, our study chose 100 pmol/mouse as the amount of Aβ injection.

**Figure 10:**
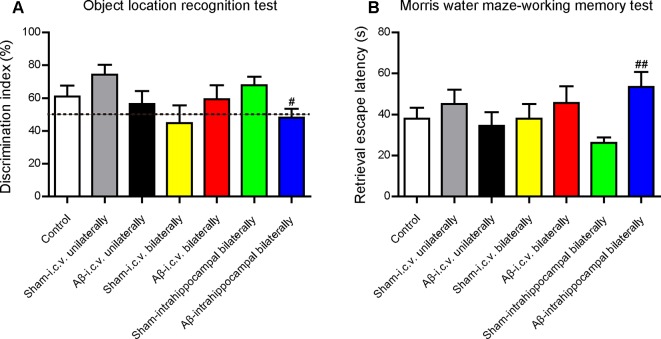
Effects of injection sites of Aβ_1–42_ oligomers on mice cognition detected by behavioral tests. **(A)** The discrimination index in test trials of object location recognition test (Mann-Whitney *U* test, n = 11–12 mice per group). **(B)** Retrieval escape latency in working memory test of Morris water maze (Mann-Whitney *U* test, n = 10–12 mice per group). Values represent mean ± SEM. ^#^ *P* < 0.05 and ^##^ *P* < 0.01 as compared to the corresponding sham-operated group. Intracerebroventricular, i.c.v.

Before the *in vivo* experiments to assess the effects of CSA on learning and memory, we operated a preliminary experiment to choose a suitable injection site to build the animal model. Unilateral injection into the ventricle (−0.2 mm AP, 1.0 mm ML, 2.5 mm DV), bilateral injection into the ventricle (−0.2 mm AP, ± 1.0 mm ML, 2.5 mm DV) and bilateral injection into the CA1 region of hippocampus (−2.0 mm AP, ± 1.5 mm ML, 1.5 mm DV) were compared under the same volume of Aβ_1–42_ oligomers solution (100 pmol/mouse). Learning and memory capacity was assessed using OLR, open filed and MWM tests following nearly the same experimental schedule (**Figure 1**). Mice in all groups had similar locomotor activity without significant difference. Compared with the mice in the corresponding sham-operated group, mice in Aβ_1–42_ oligomers-injected group performed differently in the OLR test and working memory test in MWM (**Figure 10**). Only into the bilateral CA1 region of hippocampus could Aβ_1–42_ oligomers cause a significant decrease of discrimination index in recognition test (*P* = 0.021) and a marked increase of retrieval escape latency in working memory test (*P* = 0.003). The results of intracerebroventricular injection are inconsistent with some outcomes published before (Youssef et al., 2008; Figueiredo et al., 2013; Brito-Moreira et al., 2017). These inconsistent results might be due to different amounts of injected Aβ oligomers or mice species which were summarized in a review we published previously (Wang et al., 2018). In our experimental environment, Aβ_1–42_ oligomers (100 pmol/mouse) injected bilaterally into the CA1 region of hippocampus could impair cognition successfully and be used for evaluating the role of CSA in learning and memory.

Accumulating evidence shows that soluble Aβ oligomers are more toxic than insoluble fibrils and responsible for cognitive impairment in AD pathogenesis (Dahlgren et al., 2002; Gong et al., 2003; Balducci et al., 2010; DaRocha-Souto et al., 2011). In our study, bilateral intrahippocampal injection of Aβ_1–42_ oligomers impaired the short-term spatial memory and discrimination in the OLR test, slowed down the pace of spatial learning in acquisition test of MWM, damaged working memory, and impaired the non-spatial fear-aggravated memory in passive avoidance task. These results provide additional evidence for the neurotoxic effects of Aβ_1–42_ oligomers. Compared with Aβ_1–42_ oligomers-injected group, CSA treatment significantly elevated discrimination index in the OLR test, shortened escape latency and enhanced working memory in MWM, and reduced error times in passive avoidance task, suggesting that CSA could rescue Aβ_1–42_ oligomers-induced impairment of mice learning and memory.

The Aβ oligomers hypothesis proposed by Klein (2002) suggests that early stage memory impairment in AD stems not from neuron death but rather from malfunctions in memory-specific signal transduction required for synaptic plasticity. Consistent with the hypothesis, significant neuron death was not found in the hippocampus after bilateral intrahippocampal injection of Aβ_1–42_ oligomers in our study. This result is in accordance with the report of Kasza et al. (2017). Ágnes Kasza compared the neurotoxic effects of different kinds of Aβ_1–42_ oligomers at three different aggregation concentrations (25, 75, and 200 μM) under two different aggregation times (24 and 168 h). The Aβ_1–42_ oligomers used in our study was incubated at 100 μM for 16 h, which is similar to the Aβ_1–42_ oligomers (75 μM/24 h) investigated by Ágnes Kasza. Their Aβ_1–42_ oligomers (75 μM/24 h) significantly impaired rat spatial memory in MWM and reduced long-term potentiation (LTP) levels but did not decrease the number of viable neurons significantly by Nissl staining in the hippocampus. The consistent result can also be found in the report of Yang et al. (2018). Aβ_1–42_ oligomers (100 μM/24 h) not only impaired spatial learning and memory of rats in MWM but also suppressed LTP in the DG region. However, there was no obvious difference in the number of NeuN positive cells in DG region between the Aβ_1–42_ oligomers-injected group and the control group. Therefore, bilateral intrahippocampal injection of Aβ_1–42_ oligomers (100 μM/16 h) in our study impaired learning and memory without neuron death and could successfully simulate the early stage of AD. CSA treatment had no impact on neuron death as well, suggesting that the protective effects of CSA might not be involved in the modulation of neuron death.

It has been recognized that brain inflammation involving glial cells is a prominent feature of AD (Chun et al., 2018). The activated microglia and reactive astrocytes have been observed in the brains of AD patients and animal models (Nagele et al., 2003; Heneka et al., 2005; Olabarria et al., 2010; Fan et al., 2017). Consistent with a previous study (Ledo et al., 2013), we also demonstrated that Aβ_1–42_ oligomers could induce the activation of microglia and reactivity of astrocytes in the hippocampus of mice. On one hand, the activated microglia and reactive astrocytes in Aβ_1–42_ oligomers-injected group might, in turn, stimulate the clearance of Aβ and delay the pathological progression (Wyss-Coray et al., 2003; Mandrekar et al., 2009). On the other hand, the microglial activation and astrocyte reactivity and associated inflammation could become deleterious, contributing to neurotoxicity (Hickman et al., 2008; Verkhratsky et al., 2010). In the present study, CSA significantly reduced the level of Aβ and attenuated Aβ_1–42_ oligomers-induced activation of microglia and reactivity of astrocytes in the hippocampus. These results suggest that CSA could stimulate the clearance of Aβ and the underlying mechanism needs to be explored in further studies. Downregulation of the microglial activation and astrocyte reactivity by CSA might be due to the decreasing level of Aβ or the anti-inflammatory effects of CSA (Patel and Bhutani, 2014; Huang et al., 2016; Schuster et al., 2016).

Glu and GABA dysregulation also contribute to the progression of AD. Attenuated Glu transport and decreased GABA levels were observed previously in the brains of AD patients (Masliah et al., 1996; Bai et al., 2015). We found elevated Glu levels and decreased GABA levels in the hippocampus of Aβ_1–42_ oligomers-injected group. Many factors contribute to this aberrant rise in Glu levels. Proinflammatory mediators potentially released from activated microglia in pathological conditions have been shown to influence the efficiency of astrocytic Glu uptake (Zhao et al., 2004). Aβ downregulates Glu uptake capacity of astrocytes and thereby induces a dysfunctional extracellular Glu clearance (Matos et al., 2008). The Glu released from neurons cannot be cleared from the synapse by astrocytes which increases the spillover of Glu from synapses to extrasynaptic space (Pal, 2018). Aβ also induces the release of microglial and astrocytic Glu, which in turn activates extrasynaptic NMDA receptors (Noda et al., 1999; Talantova et al., 2013). CSA treatment decreased the high levels of Glu but increased the low levels of GABA in the hippocampus. The prohibition against Aβ_1–42_ oligomers-induced high levels of Glu by CSA might be owing to its clearance of Aβ or prevention of microglial activation and astrocyte reactivity.

Synapse loss, a structural correlate involved in cognitive decline, is an early event in the progression of AD (Scheff et al., 2006). NMDARs play an essential role in synaptic function and plasticity (Collingridge and Singer, 1990) but can also be harmful (Waxman and Lynch, 2005). An explanation for the ‘NMDARs paradox’ revealed recently is the location of NMDARs: the activation of synaptic NMDARs initiates plasticity and stimulates cell survival, whereas the activation of extrasynaptic NMDARs promotes cell death and thus contributes to the etiology of AD (Wang and Reddy, 2017). Numerous studies have demonstrated that Aβ oligomers decrease synaptic NMDARs expression (Geng et al., 2013) but activate extrasynaptic GluN2B (Li et al., 2011; Talantova et al., 2013). In our study, Aβ_1–42_ oligomers increased the expression of GluN2B but decreased the expression of pCREB/CREB and BDNF in the hippocampus. Because synaptic NMDARs induce CREB activity and BDNF gene expression whereas extrasynaptic NMDARs lead to dephosphorylation of CREB that blocks induction of BDNF expression (Hardingham et al., 2002), it can be concluded that the increasing levels of GluN2B induced by Aβ_1–42_ oligomers might mainly occur at the extrasynaptic membrane. Based on the fact that PKA activity is necessary and sufficient for synaptic clustering of NMDARs (Crump et al., 2001), the decreasing levels of PKA c-α, a kind of catalytic subunit of PKA, in Aβ oligomers-injected group indicate that Aβ_1–42_ oligomers might inhibit PKA activity and thus downregulate synaptic NMDARs. Taken together, extrasynaptic GluN2B might be upregulated while synaptic NMDARs might be downregulated after bilateral intrahippocampal injection of Aβ_1–42_ oligomers in the present study. CSA treatment reversed the Aβ_1–42_ oligomers-induced excessive expression of GluN2B, decreased the high level of Glu, enhanced the downstream PKA/CREB/BDNF/TrkB signaling pathway and might decrease extrasynaptic NMDARs but increase synaptic NMDARs activity. More direct evidence of CSA effects on extrasynaptic or synaptic NMDARs needs to be provided by an electrophysiological method using MK-801 to block active synaptic NMDARs in the further experiment (Li et al., 2011). No significant difference of GluN1 levels was observed among all groups, suggesting that the effects of Aβ_1–42_ oligomers or CSA did not involve in the modulation of GluN1 total expression.

## Conclusions

In summary, our study provides a validated animal model to simulate the early stage of AD by bilateral injection of Aβ_1–42_ oligomers into the CA1 region of the hippocampus. CSA effectively improved the cognitive impairment induced by Aβ_1–42_ oligomers. Its cognitive enhancing effects might be mediated by clearing Aβ, preventing microglial activation and astrocyte reactivity, reversing Glu and GABA dyshomeostasis, inhibiting excessive expression of GluN2B and enhancing the downstream PKA/CREB/BDNF/TrkB signaling pathway in the hippocampus. Our study provides the possibility for CSA to be a potential therapeutic agent at the early stage of AD.

## Data Availability

All datasets generated for this study are included in the manuscript/**Supplementary Files**.

## Ethics Statement

The animal study was reviewed and approved by the Animal Ethical Committee at the Institute of Medicinal Plant Development, Chinese Academy of Medical Sciences.

## Author Contributions

QC, R-LP, X-ML, Y-HL and L-SW participated in research design. L-SW, XT, Y-FZ and M-DZ conducted experiments and analyzed data. L-SW and QC wrote the manuscript. All authors reviewed the manuscript.

## Funding

This work was supported by the CAMS Innovation Fund for Medical Sciences (CIFMS, 2016-I2M-1-012), the National S&T Major Projects for New Drug Innovation and Development (2017ZX09301029) and the National Key R&D Program of China (2018YFC1602105).

## Conflict of Interest Statement

The authors declare that the research was conducted in the absence of any commercial or financial relationships that could be construed as a potential conflict of interest.

## Abbreviations

CSA, cajaninstilbene acid; AD, Alzheimer’s disease; Aβ, amyloid β peptide; MWM, Morris water maze; Glu, glutamate; GABA, γ-aminobutyric acid; NMDARs, N-methyl-D-aspartate receptors; CREB, cAMP response element binding protein; PKA, cAMP-dependent protein kinase; BDNF, brain-derived neurotrophic factor; TrkB, tropomyosin receptor kinase B; sECC, stilbenes containing extract-fraction from Cajanus cajan (L.) leaves; HFIP, 1,1,1,3,3,3-hexafluoro-2-propanol; DMSO, dimethyl sulfoxide; PBS, phosphate buffered saline; AFM, atomic force microscopy; CMC-Na, carboxymethylcellulose sodium; OLR, object location recognition; PFA, paraformaldehyde; IOD, integrated optical density; MRM, multiple reactions monitoring; ANOVA, analysis of variance; LSD, least significant difference; LTP, long-term potentiation.
