# Supplementary material for: Cajaninstilbene Acid Ameliorates Cognitive Impairment Induced by Intrahippocampal Injection of Amyloid-β1–42 Oligomers
Source: Front Pharmacol. 2019 Sep 24;10:1084. doi: 10.3389/fphar.2019.01084 (PMC6798059; doi:10.3389/fphar.2019.01084)
Supplement: Supplementary file 1 [file DataSheet_1.zip › Supplemental figures of western blot.docx]

**The full, untruncated images of the gels in our manuscript**


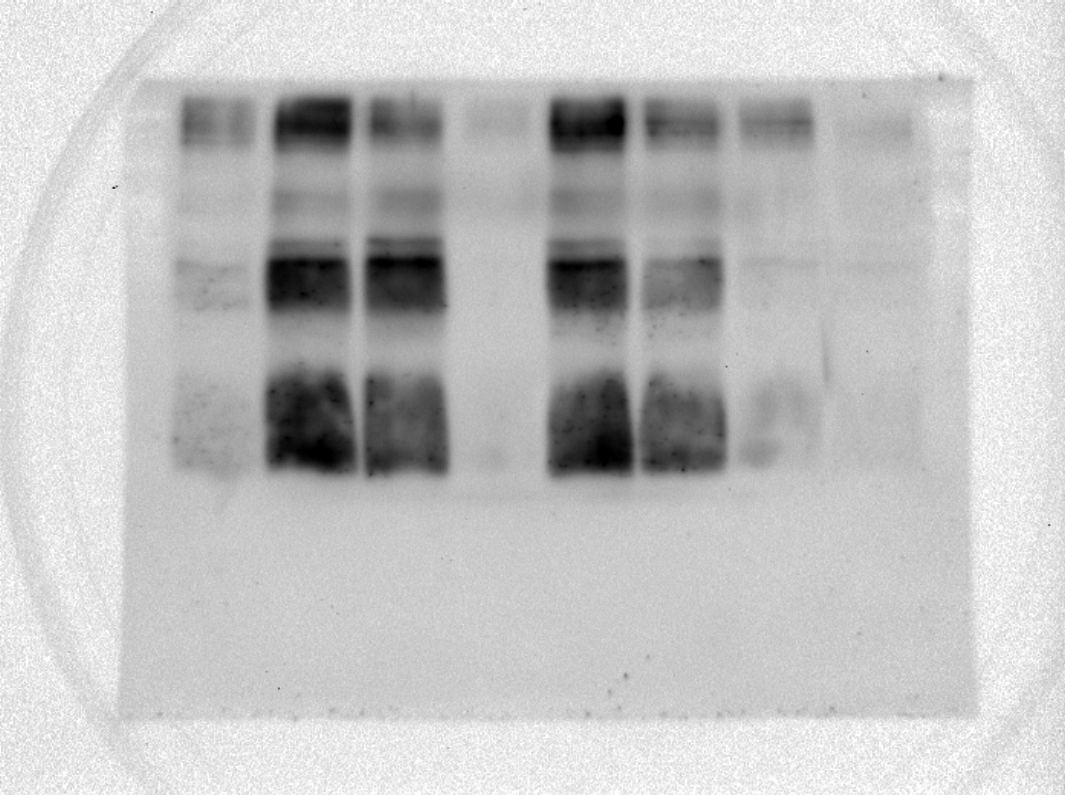


**Figure 2B**


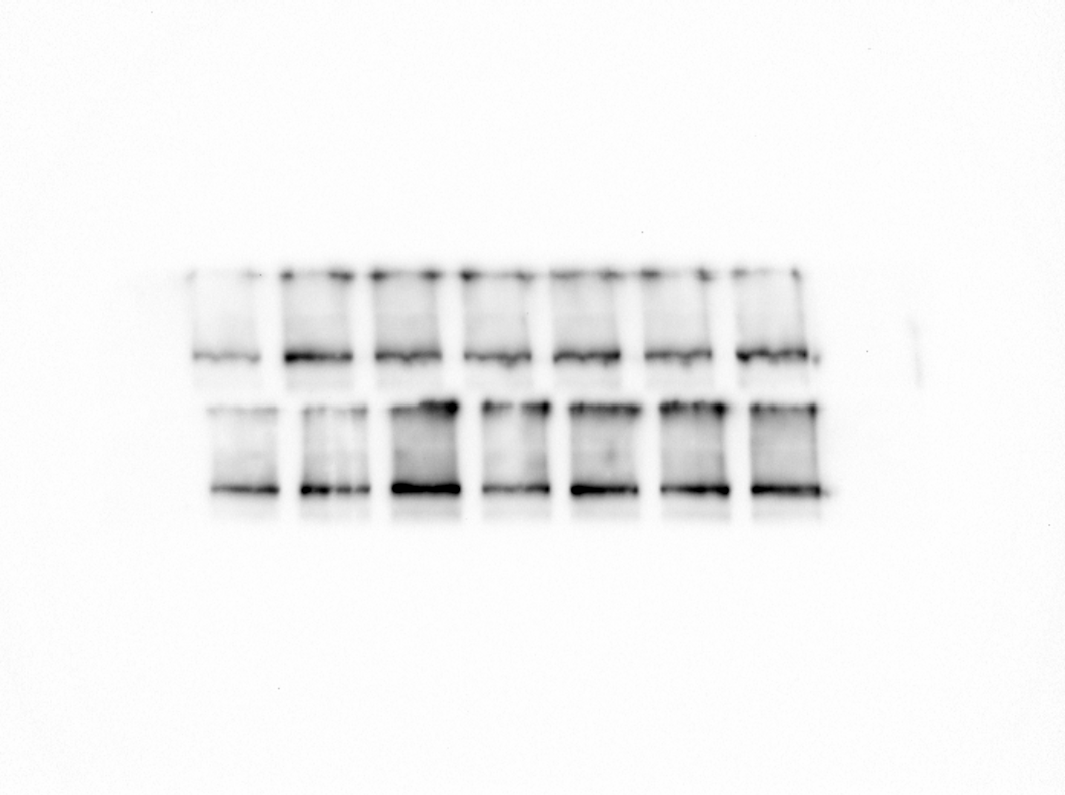

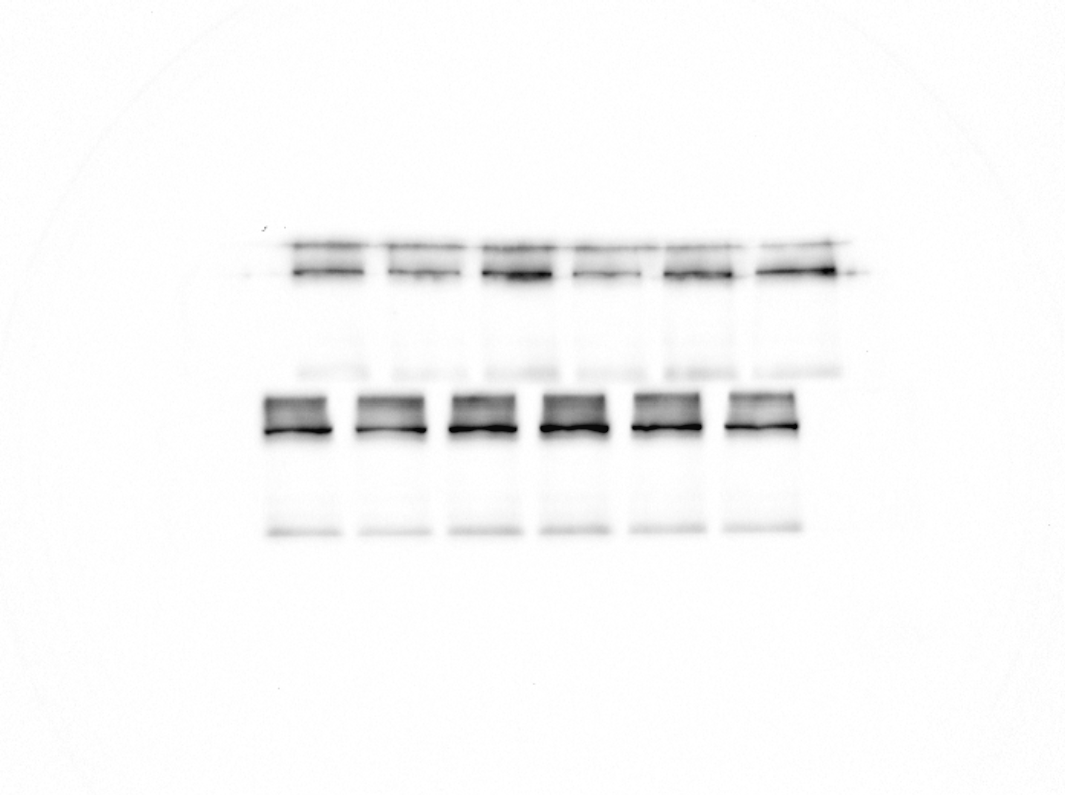


**Figure 8A GluN2B**


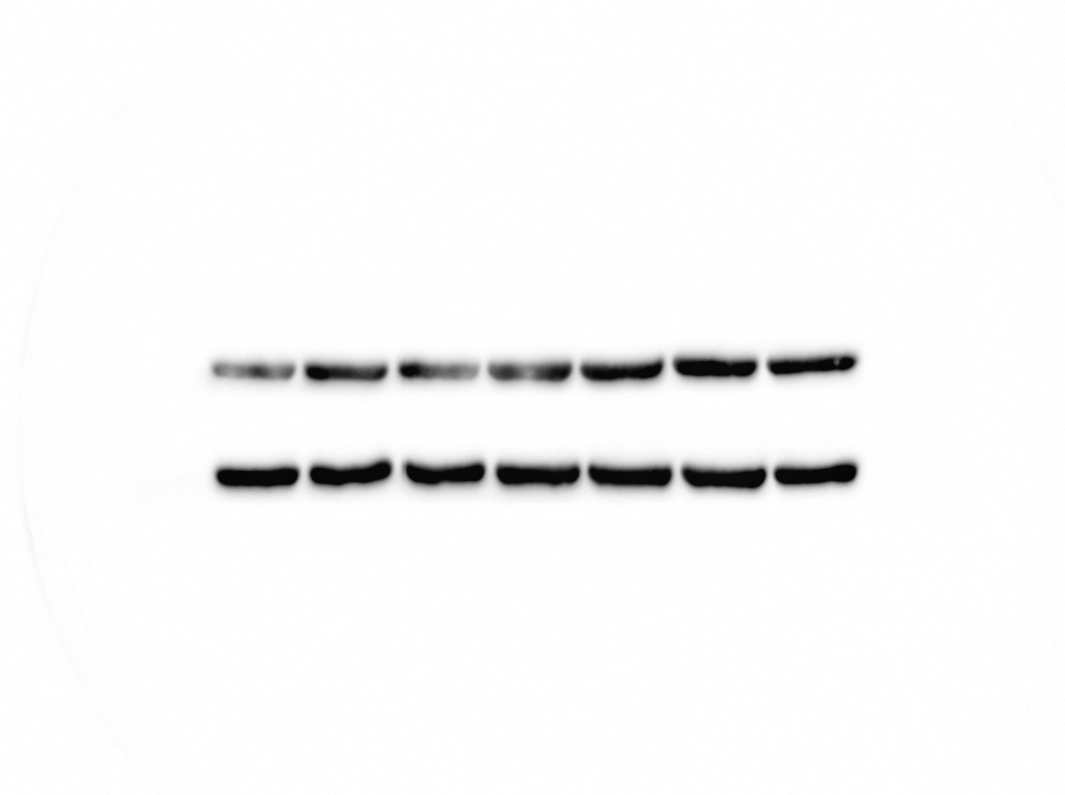


**Figure 8A β-actin**


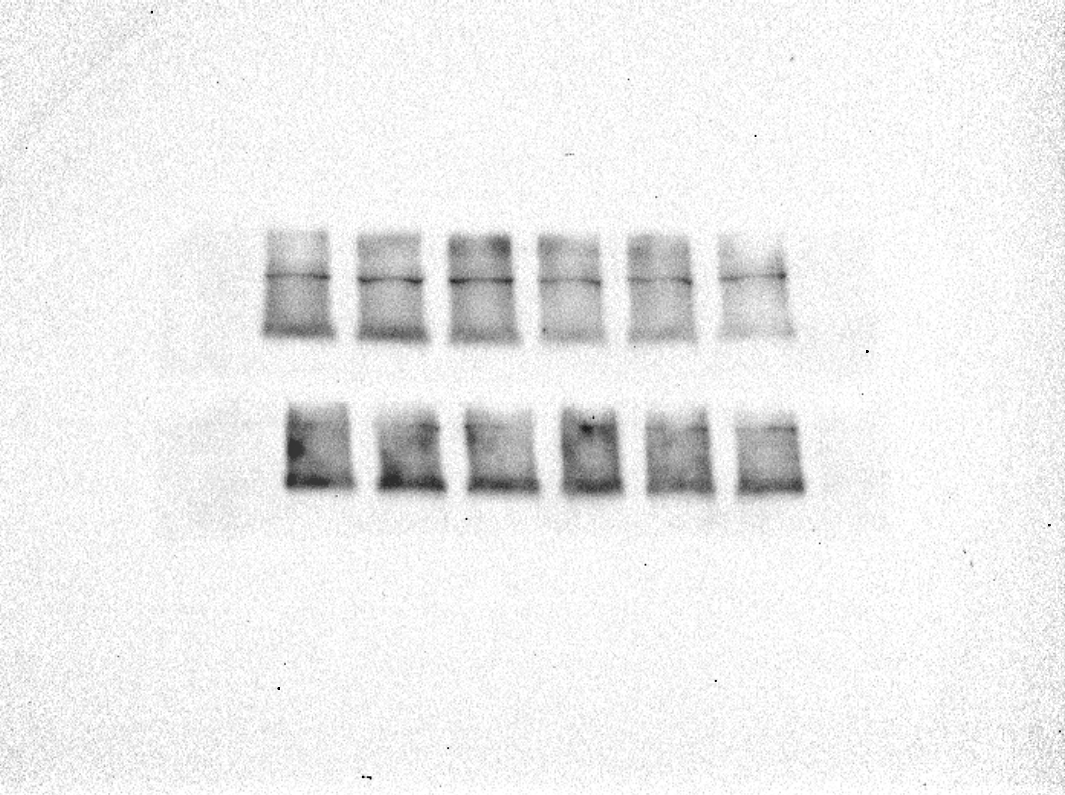


**Figure 8B GluN1**


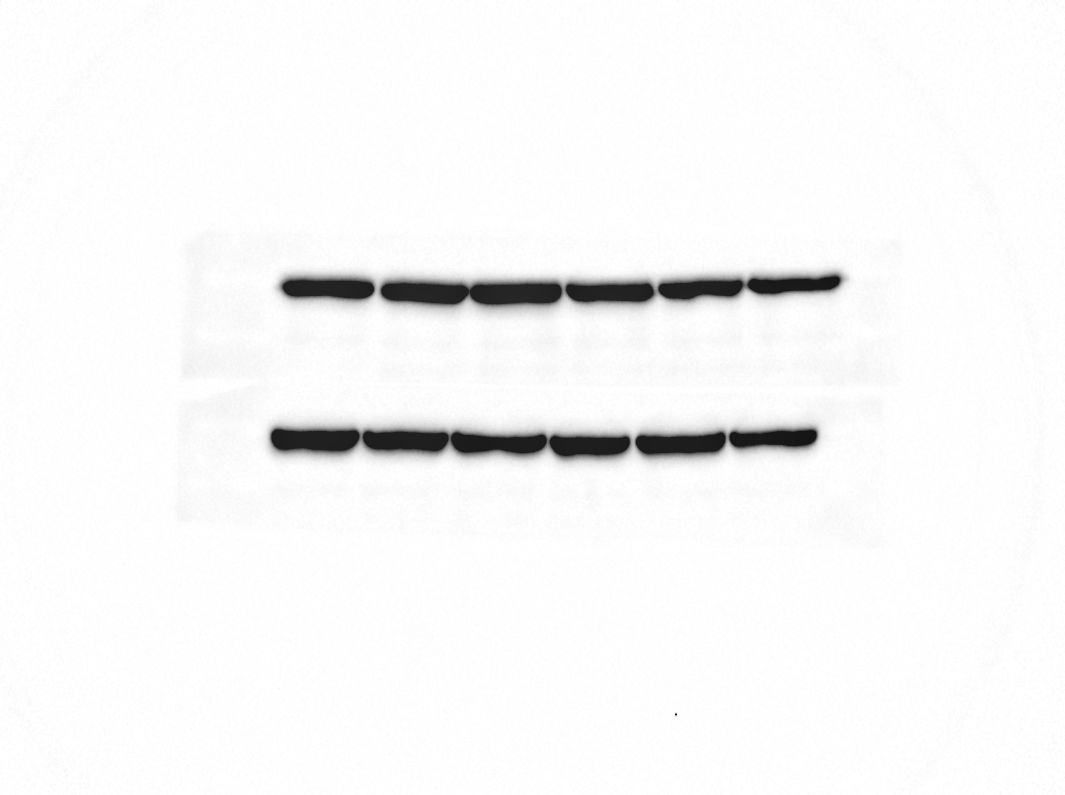


**Figure 8B β-actin**


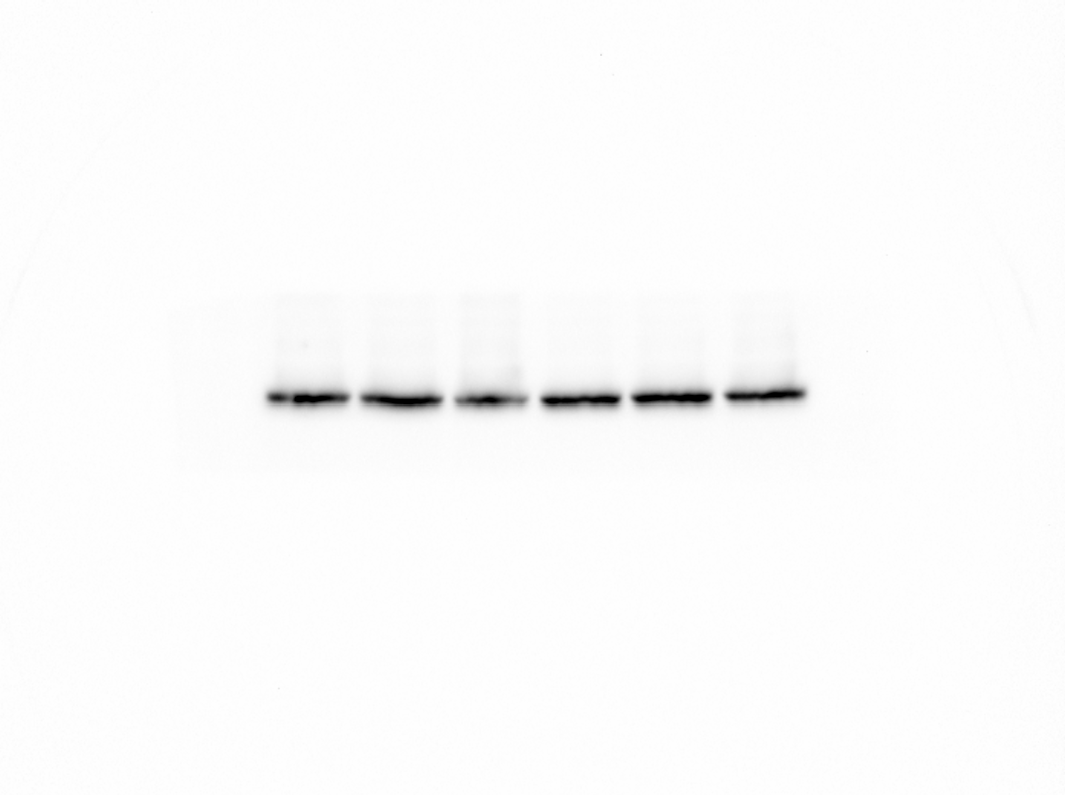


**Figure 8C PKA**


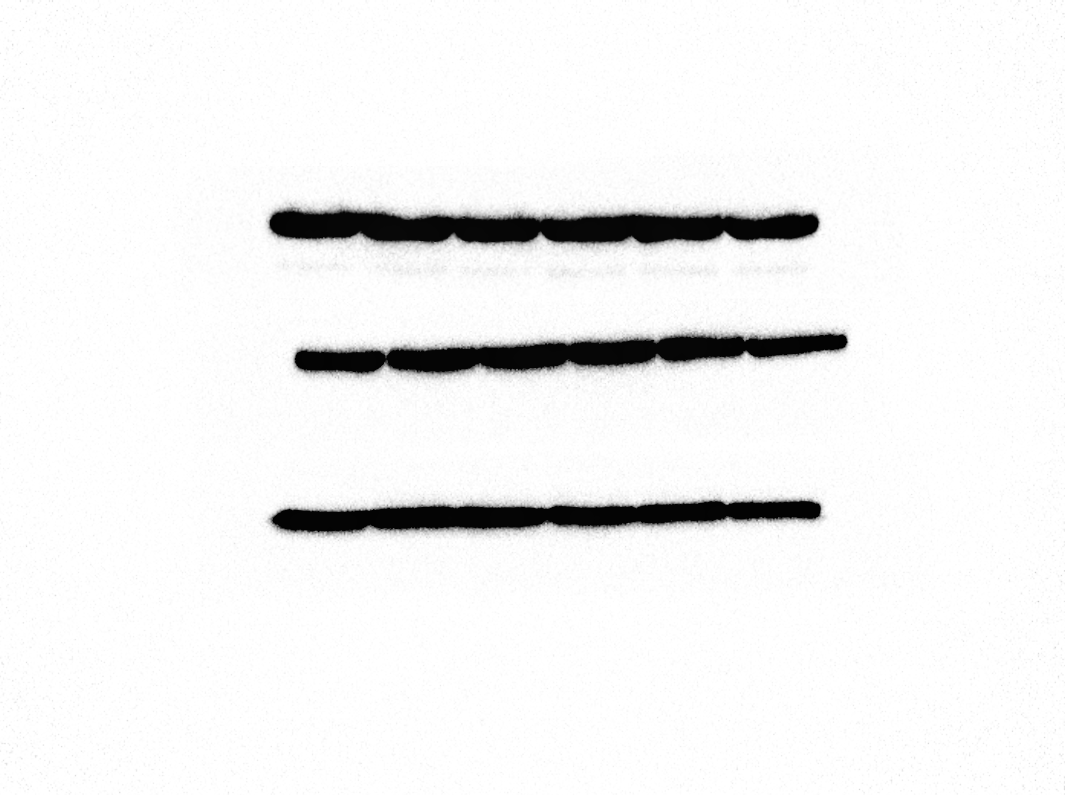


**Figure 8C β-actin**


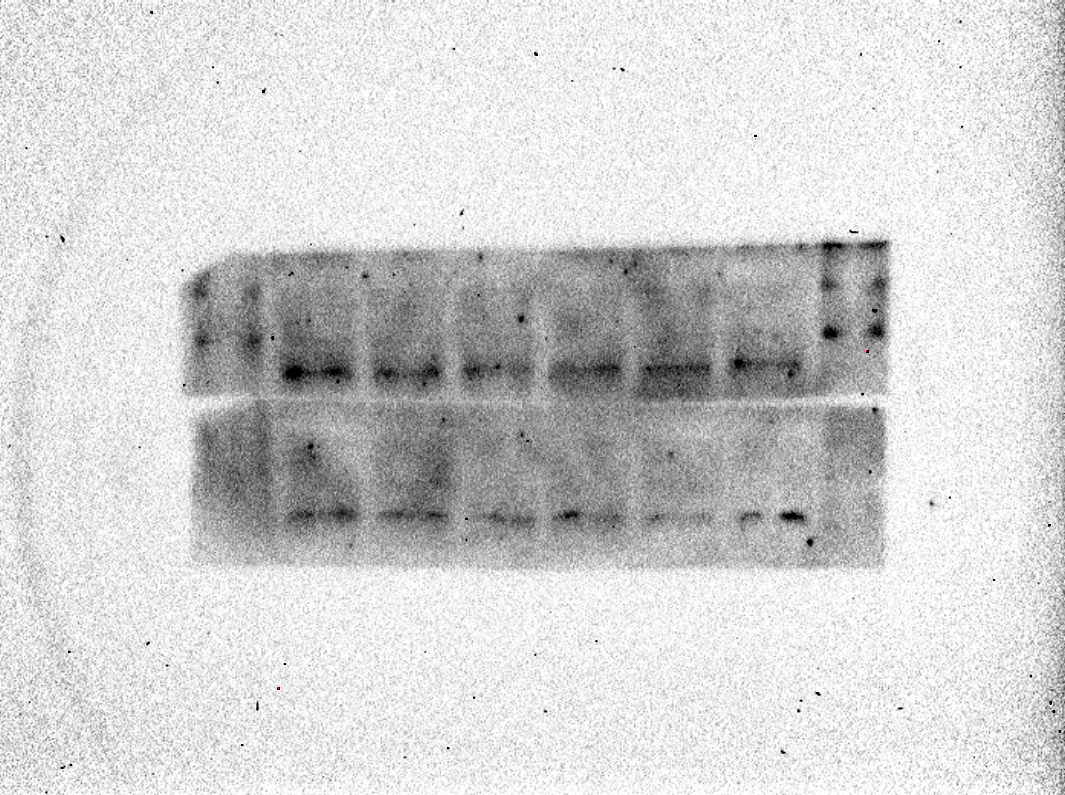


**Figure 8D p-CREB**


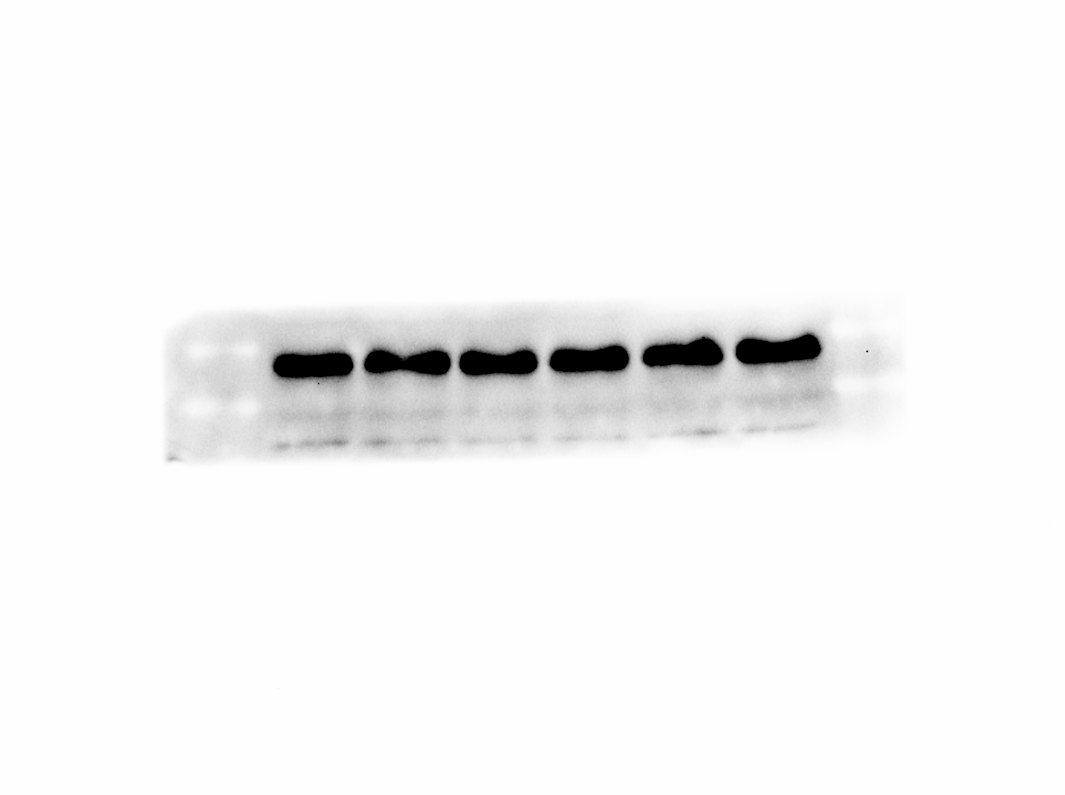


**Figure 8D CREB**


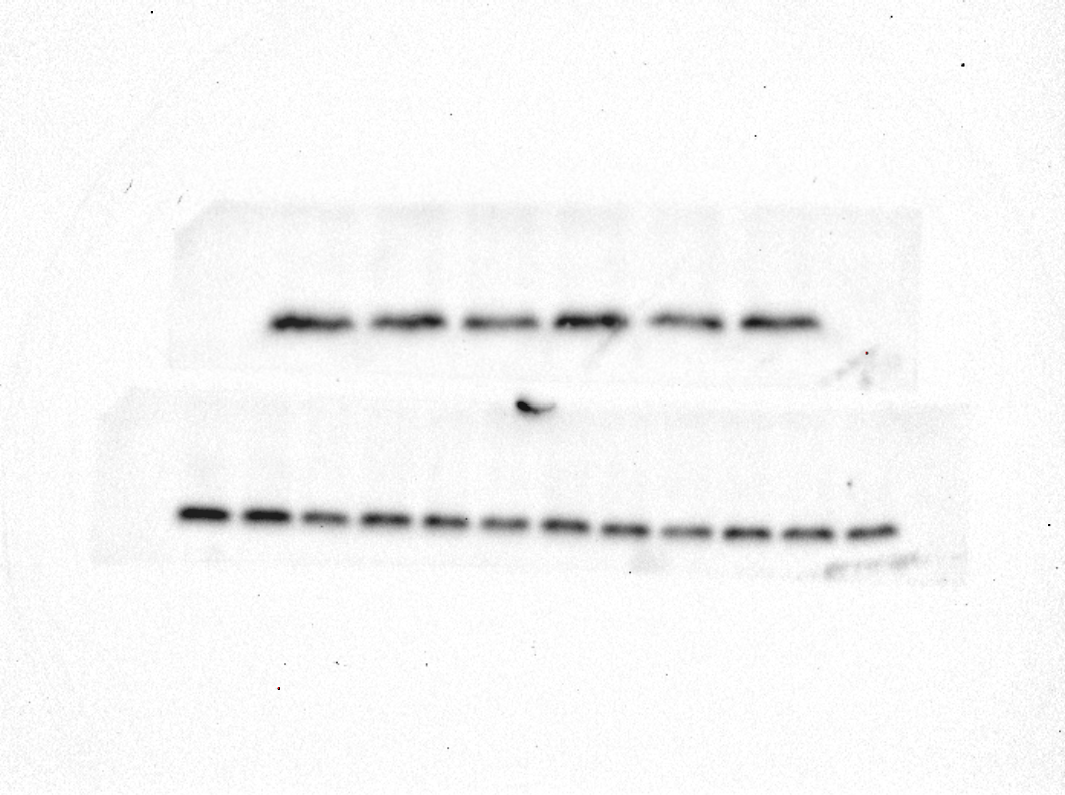


**Figure 8E BDNF**


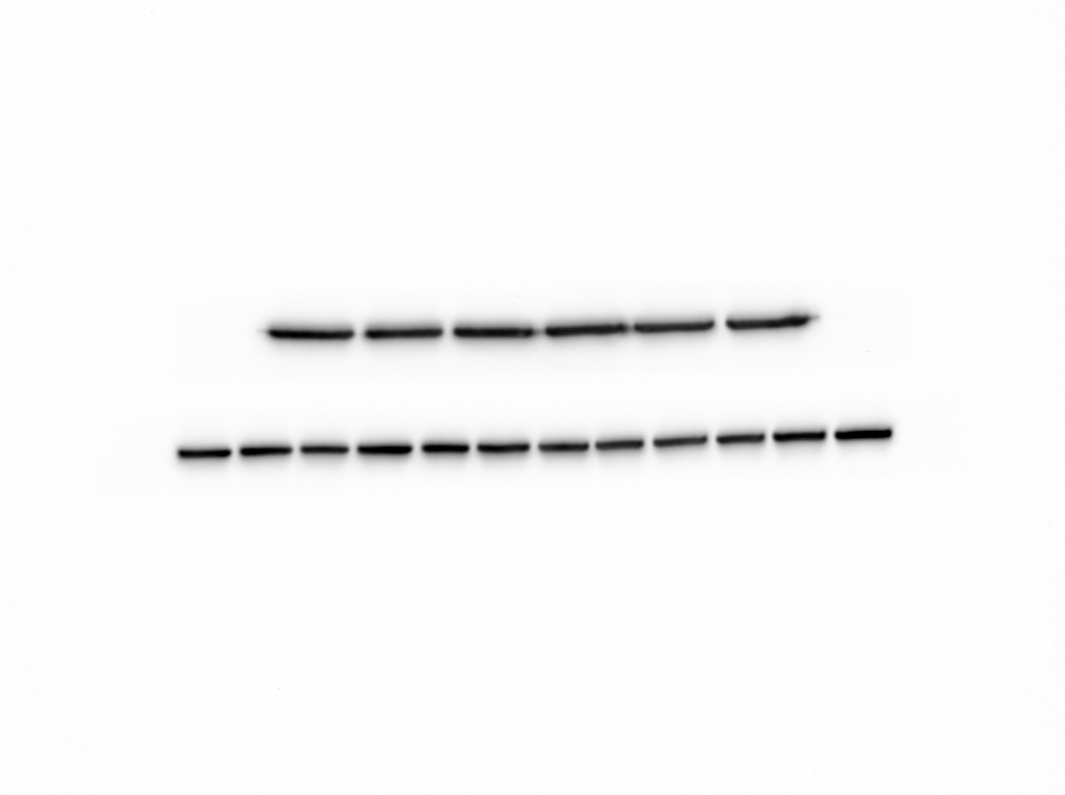


**Figure 8E β-actin**


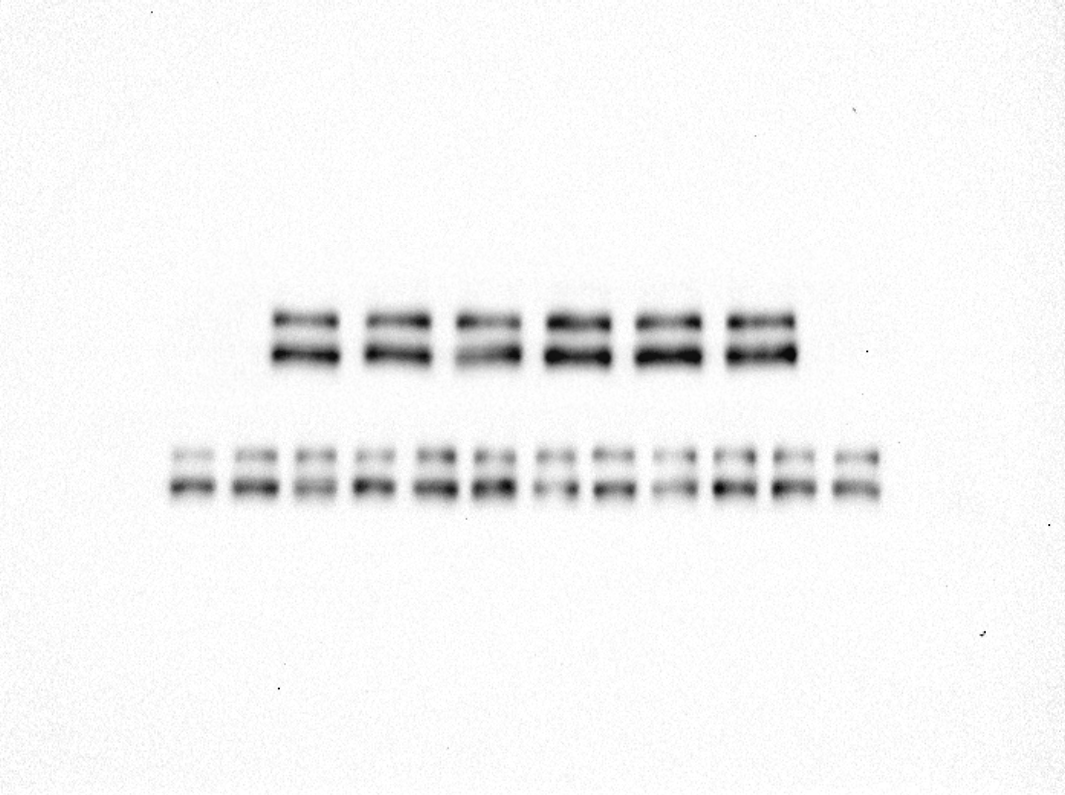


**Figure 8F TrkB**


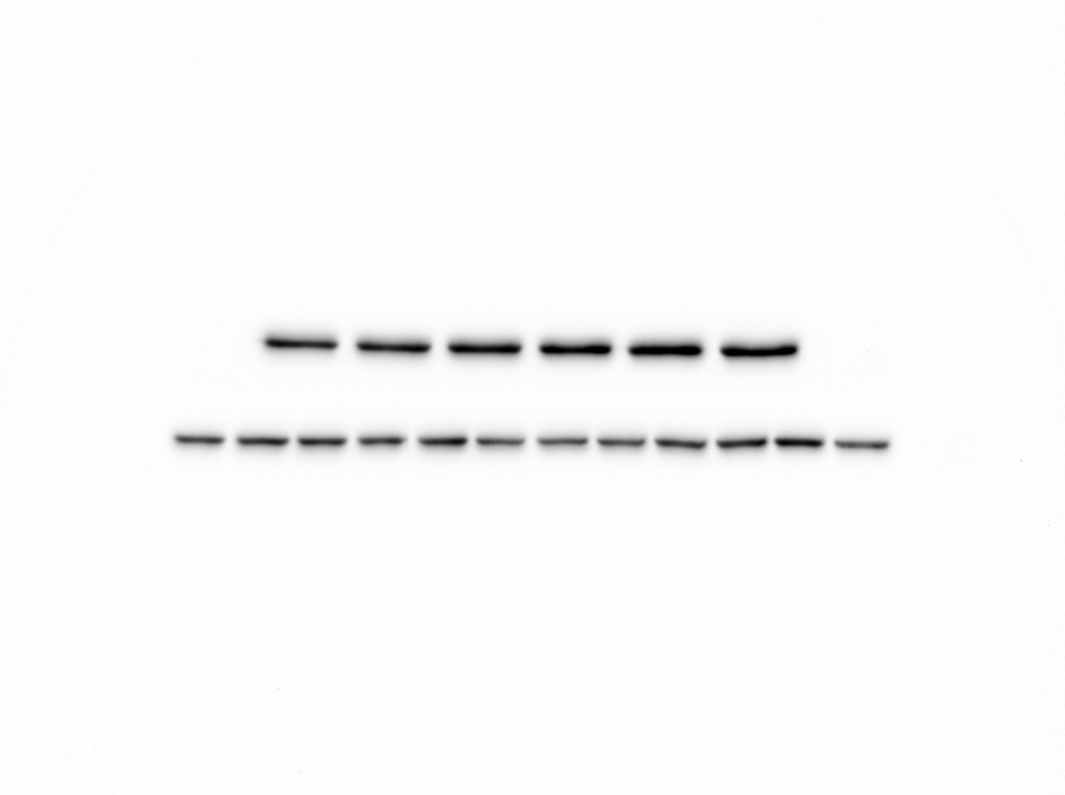


**Figure 8F β-actin**
